# Organizational health factors: multidimensional insights into occupational stress in higher education

**DOI:** 10.3389/fpsyg.2025.1715167

**Published:** 2026-01-21

**Authors:** Isabel Souto, Anabela Sousa Pereira, Elisabeth Brito, Paulo Alves, Daniel Marrinhas

**Affiliations:** 1 Department of Psychology, Autonomous University of Lisbon, Lisbon, Portugal; 2 Department of Education and Psychology, University of Aveiro, Aveiro, Portugal; 3 Department of Psychology, University of Évora, Évora, Portugal; 4 Center for Research in Education and Psychology (CIEP), University of Évora, Évora, Portugal; 5 School of Technology and Management of Águeda, University of Aveiro, Aveiro, Portugal; 6 Research Unit on Governance, Competitiveness and Public Policies, (GOVCOPP), University of Aveiro, Aveiro, Portugal; 7 Piaget Research Center for Ecological Human Development, Lisbon, Portugal

**Keywords:** burnout, COVID-19, health and well-being, higher education teachers, occupational stress, organizational health, psychosocial risk factors, resilient coping

## Abstract

**Introduction:**

Occupational stress (OS) among higher education (HE) teachers is a growing concern, intensified by the COVID-19 pandemic. This study explores OS through the Organizational Health (OH) Framework, integrating individual, organizational, and contextual factors to assess their impact on teacher Health and Well-being.

**Methods:**

A cross-sectional study was conducted with 401 Portuguese HE teachers. Data were collected via validated instruments including the Brief Resilient Coping Scale, COPSOQ III, Kessler Psychological Distress Scale, and a COVID-19-related checklist. Statistical analyses included correlations, ANOVA, and multiple regressions to identify predictive relationships.

**Results:**

Findings revealed that (i) The professional context of teachers has its own characteristics, with low levels of resilient coping, high exposure to job demands, particularly cognitive demands. Concerning the Covid-19 pandemic, fear of infecting someone and the exaggeration of information on social media emerge as the most frequent causes of fear. Satisfaction with the level of adaptation to the pandemic situation, in terms of lifestyle, was high. For 32.7% of participants, Distress values have clinical significance, as well as Sleep Problems, Burnout, Stress, and Depressive Symptoms; around 40% of responses fell into the risk category for health impact. (ii) The Health and Well-being of Higher Education teachers is influenced by the interaction of individual and organizational factors, and vice versa, while both are influenced by contextual factors, namely experiences associated with the COVID-19 pandemic. (iii) Work–family conflict and Job Demands are risk factors with a pronounced predictive effect on Health and Well-being, while Resilient Coping and Meaningfulness of work stand out for their protective effect. Satisfaction with lifestyle concerning Covid-19 emerged as a predictor in all Health and Well-being domains.

**Discussion:**

The study underscores the complex interplay between individual, organizational, and contextual factors in shaping teacher Health and Well-being. It highlights the need for targeted interventions and organizational policies that foster resilience and mitigate stress. Promoting well-being in HE requires integrated strategies addressing systemic and individual dimensions, with implications for public policy and institutional practice.

## 1 Introduction

The student population growth, driven by migratory flows and demographic changes, alongside the imperative to align education systems with the evolving demands of a labor market shaped by rapid technological evolution, has resulted in a profound global reconfiguration of Higher Education (HE) (Hart and Rodgers, 2024; Marshall et al., 2024; Zgaga et al., 2014). All of these factors required a collective effort of all involved, including HE teachers, demanding constant adjustment to accomplish professional excellence in a highly competitive and demanding setting (Ahmad et al., 2022; European Commision: European Education and Culture Executive Agency [EC: EECEA], 2018; Hart and Rodgers, 2024; Zgaga et al., 2014).

In this context, the role of HE teachers has become multifaceted, extending beyond teaching to include responsibilities such as supervising students in internships, research projects, and even engage in administrative and bureaucratic tasks (Souto et al., 2018; Sumoza Sumoza and Faneite, 2019). One of the most prominent sources of workload pressure is the tension between teaching and research, particularly within the “*publish or perish*” culture, where academic career progression is closely tied to the quantity and visibility of scientific publications (Bowering and Reed, 2021; Heng et al., 2020; Miller et al., 2011). As such, both classical and contemporary studies reveal that the noble profession of HE teacher in is increasingly exposed to a series of Psychosocial Risk Factors (PRFs) and the consequent experience of Occupational Stress (OS), whose impact on well-being is far from being an abstract or purely theoretical concern (Carvajal and Guedea, 2021). PRFs such as work overload, time constraints, longer working hours (Ahmad et al., 2022; Cladellas and Castelló, 2011), reduced autonomy and control, the ambiguity of roles or the lack of communication (Faisal et al., 2019; Souto et al., 2018, 2019; Sun et al., 2011) have emerged in the literature as factors to be particularly considered, not least because of the harmful effects on health and general well-being, as well as effects on life satisfaction, job satisfaction, and work-family balance (Carvajal and Guedea, 2021; García-Arroyo and Osca Segovia, 2019; Mahecha-Naranjo et al., 2023; Salim et al., 2023; Tai et al., 2019; Urbina-Garcia, 2020; Wu and Zhou, 2023). For example, classic studies in HE context identified that administrative overload and pressure for scientific productivity compromise the time dedicated to teaching, as well as work intensification were associated with symptoms of emotional exhaustion (Cladellas and Castelló, 2011; Seibt et al., 2012; Wiegel et al., 2016). Recent studies highlight that OS is strongly associated to physical health, as musculoskeletal or voice disorders (Tai et al., 2019), mental health problems, such as anxiety, depression and burnout (Carvajal and Guedea, 2021; Mahecha-Naranjo et al., 2023; Salim et al., 2023; Urbina-Garcia, 2020), as well as deterioration of the balance between personal and professional life (Bowering and Reed, 2021).

Furthermore, as the demands placed on HE teachers continue to intensify in an ever-evolving academic landscape (Springer et al., 2023; Urbanek, 2021; Zgaga et al., 2014), the Covid-19 pandemic crisis added another layer of complexity. This global crisis led to a rapid and unprecedented transition from face-to-face to distance and online teaching, forcing teachers to adapt abruptly to new technologies and e-pedagogies without the time or training for such a change (Coman et al., 2020; Scherer et al., 2021; Virtanen and Parpala, 2023). If, on the one hand, the encounter with new technologies and teaching methods can trigger a variety of attitudes (Marrinhas et al., 2023; Meishar-Tal and Levenberg, 2021; Scherer et al., 2021), on the other hand, as teachers grappled with the new challenges posed by the pandemic, the impact of OS on mental health became an even more prominent concern (Almhdawi et al., 2021; Leitao and Capuzzo, 2021), which led teachers to reassess their role in the academic setting, including the intention to leave the profession (Scherer et al., 2021; Virtanen and Parpala, 2023). The challenges experienced were manifold and multifaceted, encompassing several domains, namely, the aforementioned demands inherent in adapting to distance learning and interruptions in research efforts (Marrinhas et al., 2023; Meishar-Tal and Levenberg, 2021; Qurat-Ul-ain, 2021), experiences of fears and anxieties associated with the pandemic (Naveed et al., 2022), as well as the balance between professional and personal life, particularly in teleworking (Cluver et al., 2020; Horton and Jacobs, 2022; Naveed et al., 2022). Thus, the pandemic has increased the need to cultivate a flexible, supportive, and caring work environment for these professionals, with the aim of mitigating their exposure to OS, both in face-to-face and remote work environments, and during the transition between work modalities (Horton and Jacobs, 2022; Marrinhas et al., 2023; Scherer et al., 2021).

To address the global risk posed by exposure to OS, it is imperative to comprehend its nature, sources, and consequences (European Agency for Safety and Health at Work [EU-OSHA], 2018; International Labour Organization, 2016; Wolf and Prüss-Ustün, 2018). In the context of HE, it is important to acknowledge the persistent emphasis on opportunities for continuous professional development and lifelong learning, which are aspects that must be taken into consideration (Ahmad et al., 2022; Faisal et al., 2019). Additionally, the way individuals perceive their own ability to deal with circumstances where desired outcomes are difficult to achieve, i.e., their perception of self-efficacy, is a key component of the self-regulation system, serving as a crucial determinant of behavioral choices and the initiation and persistence of actions. Thus, individuals with high beliefs in self-efficacy are more likely to approach difficult tasks with confidence, persistence, and resilience (Bandura, 1997, 2012). Note that, it has been observed that a low level of pedagogical self-efficacy, particularly in the context of remote and online teaching, is associated with diminished job satisfaction and perceived support from the work environment (Scherer et al., 2021). In turn, a strong perception of self-efficacy positively influences the assessment of SO sources and the respective response strategies, leading the individual to perceive stress factors as challenges rather than threats, also acting as a buffer against the negative impact of SO or even burnout (Hirschle and Gondim, 2020; Salanova et al., 2003; Schwarzer and Hallum, 2008; Sonnentag, 2015; Stajkovic and Luthans, 1998). In addition, the use of resilient coping strategies are considered adaptive responses that promote psychological well-being and effective stress management (Denckla et al., 2020; Pais Ribeiro and Morais, 2010; Southwick et al., 2014). However, with regard to HE, the strength and consistency of this relationship remain unclear, as empirical evidence is still emerging and sometimes inconclusive (Chukwuemeka et al., 2023; García-Arroyo and Osca Segovia, 2019; Springer et al., 2023). Moreover, it is still largely unknown which specific adaptative coping strategies are most commonly employed by academics to manage the complex demands of their professional roles (Urbina-Garcia, 2020). In contrast, maladaptive coping strategies, such as increased consumption of licit and illicit psychoactive substances, alcohol, and tobacco, have been reported in response to elevated stress levels among academic professionals, raising concerns about long-term health and well-being (Filho et al., 2019; Pereira et al., 2022; Vieira et al., 2021; Wiegel et al., 2016).

Additionally, the impact of OS exposure also extends to the immediate teaching environment, for example, through reduced commitment to teaching, with tangible consequences for the quality of education provided to students, but also to aspects of research and general professional and organizational engagement (Ngirande, 2021; Valdiviezo and Guevara, 2022, 2022; Virtanen and Parpala, 2023; Wang et al., 2020; Xu et al., 2023), with foreseeable effects on the overall success of educational institutions. Since education is the cornerstone of the knowledge and skills needed for the jobs of the future, there may also be latent effects on the development and innovation of society (Belwal et al., 2023; Singh and Ryhal, 2021).

Although there is growing interest in the well-being of workers, the sample of HE teachers has not yet been thoroughly investigated (Springer et al., 2023), which is also evident in the Portuguese context. HE in Portugal has faced increasing challenges, driven by structural reforms such as the Bologna Process, which began in 1999 with the signing of the Bologna Declaration by 29 European countries (European Commision [EC], 2022). This transformation aimed not only to modernize curricula and promote academic mobility, but also to adapt teaching methods, encouraging more student-centered approaches, such as problem-based learning, over traditional one-way teaching (Curaj et al., 2024; Gomes et al., 2016; Hernandez-Encuentra and Sanchez-Carbonell, 2005; Kolmos, 2009; Tiernan, 2024).

In Portugal, teaching duties are also combined with scientific research, student supervision, managerial duties, and participation in academic projects and networks (Souto et al., 2018). As the number of students has been increasing, with more heterogeneous classes, the added challenges in pedagogical management and the adaptation of teaching methodologies are also a reality (Hauschildt, 2024). In addition, there is also pressure to publish in indexed journals, obtain competitive funding, and meet institutional performance indicators (Veiga et al., 2014). On the other hand, the limited research within the Portuguese HE context highlights high levels of psychological distress, associated with various PRFs in teachers’ work environments (Souto et al., 2018, 2019), whose negative impact on health, particularly burnout, is undeniable (Cardoso, 2002; Teles et al., 2020). The most immediate impact of the pandemic is also documented among HE teachers and researchers, both in terms of exposure to stress in general and techno stress, with regard to its consequences on health, specifically burnout rates (Marrinhas et al., 2023; Teles et al., 2020). Although the excellence of these studies is recognized, they are not without some limitations in the current context, such as the fact that they were mostly conducted in a pre-pandemic context.

In summary, the phenomenon of OS in the context of HE is complex and multifaceted. Nevertheless, few studies have examined the antecedents of OS among HE teachers from an integrated perspective. The interconnection between individual, organizational factors and well-being are often neglected in thematic studies, despite the general consensus that they are essential to guide the implementation of effective prevention and health promotion programs (Cassar et al., 2020; Cotton and Hart, 2003; Kumar and Kirti, 2023; Popov et al., 2017; Singh and Jha, 2021; Thanem and Elraz, 2022).

For this reason, the present paper adopts the Organizational Health (OH) Framework, which introduces a unique perspective on the OS (Hart and Cooper, 2001). The OH Framework posits that an organization is not just a collection of isolated individuals and processes, but includes multiple factors (individual and organizational) which interact for both the health and well-being of the workers, as well as the organization. Furthermore, the various elements that make up the organizational system exist/interact within a broader environmental context. Consequently, factors from the broader environment can also generate emergent effects that shape the overall behavior of the organization, influencing all the underlying levels and processes (Hart and Cooper, 2001; Thanem and Elraz, 2022).

It should be noted that the author of this framework proposes some dimensions that constitute the first levels of analysis, namely individual characteristics (personality), coping processes, and organizational climate (which includes positive and negative work experiences), considering that these domains are likely to contribute to distress levels, affect job satisfaction or employee morale, and therefore affect employee Health and Well-being. However, he also cautions that these are not the only dimensions to be analyzed. In other words, the OH Framework, in its heuristic form, does not provide an exhaustive list or delimitation of the set of variables central to OS/organizational health, but has a high capacity for adjustment and flexibility (Cotton and Hart, 2003; P. M. Hart and Cooper, 2001; Kumar and Kirti, 2023; Singh and Jha, 2021).

In short, the OH Framework is a model that combines the various structures and processes inherent in the OS phenomenon, the management of which is the key to reducing stress and increasing job satisfaction, health and well-being and performance (Bauer and Jenny, 2017; Cotton and Hart, 2003; Hart and Cooper, 2001; Lee et al., 2023). As such, based on this theoretical framework, we established the conceptual framework that forms the central foundation of this study. Using the OH Framework in its heuristic form, we generated hypotheses about the mechanisms and explanatory variables of OS to be tested, focusing on (i) individual factors, which include Resilient Coping and Self-Efficacy, (ii) organizational factors, emphasizing Psychosocial Risk Factors (PRFs) as factors of work content or context that can generate OS, (iii) contextual factors, namely experiences associated with the Covid-19 pandemic. We speculate that the interaction of these factors is crucial for maintaining (iv) well-being factors, notably Quality of Life (QoL), Distress, and Health Factors in this domain.

This paper aims to test the premise that individual-organization-context relationships form a dynamic set that is integrated into OS and, therefore, relevant to the maintenance of individual and organizational well-being of HE teachers. In this exploration, we delve into the complex perspective of OS in HE teachers, examining stress factors as a process, implementing an integrated approach with the theoretical framework of OH. Specifically, we sought to answer (in an integrated and multidimensional way) the following research questions:

*Question 1:* How is the work context of HE teachers characterized, considering the OH Framework?

*Question 2:* What is the relationship between OH Factors, and what is their effect on the Health and Well-being of HE teachers?

## 2 Materials and methods

### 2.1 Study design

This study is part of a doctoral research project that examined OS among HE teachers using a holistic approach that considers individual, organizational, and contextual factors, as posted on OH Framework. The project aimed to develop tools to support and promote teachers’ health and well-being. The full study is available at (*reference omitted for blind review purposes*). This research presents, for the first time, descriptive, correlational, and predictive factors related to health and well-being during and after the COVID-19 pandemic.

### 2.2 Participants

The empirical study involved 401 Portuguese HE teachers aged between 25 and 70 years (M = 52.4, SD = 8.97). The sample consisted of 137 male participants (34.2%) and 261 female participants (65.1%). It is worth mentioning that one participant identified their gender as “other,” i.e., Non-binary (NB, 0.2%), and two participants preferred not to provide gender information (NI, 0.5%).

Regarding socio-professional characteristics, the total work experience ranged from <1 to 43 years (M = 19.8, SD = 11.578). The teachers worked in public (*n* = 354, 88.3%), private (*n* = 40, 10.0%) or mixed (*n* = 7, 1.7%) establishments, in teaching modality of polytechnic (*n* = 238, 59.4%), university (*n* = 133, 33.2%) or both (*n* = 29, 7.2%).

The data on the target population, available from the Portuguese *Directorate-General for Education and Science Statistics*^1^ (DGEEC, 2022), refers to the 2021/22 academic year, with a total of *n* = 38,667 HE teachers in Portugal. To calculate the sample size, we used a significance level of 0.05, a confidence level of 95% and a sampling error of 5%, applied to the formula for finite populations. The sample obtained (*n* = 401), corresponds to a sampling error of ≈ 4.9%, as such, the total sample is considered representative.

### 2.3 Materials

**Figure 1:**
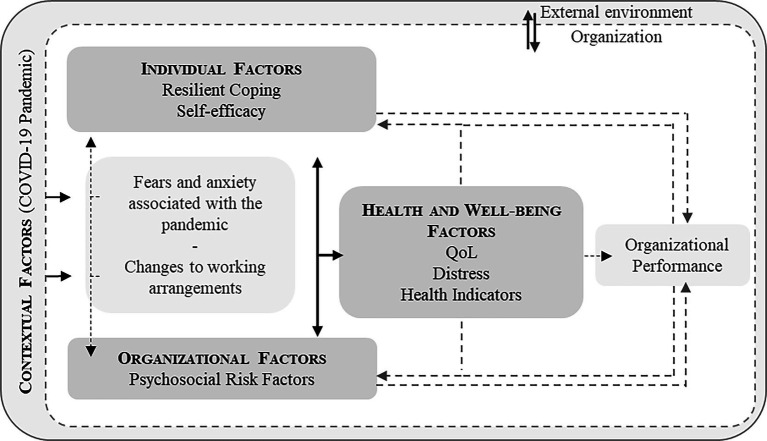
Integration of the variables selected in compliance with the Organizational Health Framework, in heuristic form (adapted from Hart and Cooper, 2001).

In this study, data collection aimed to identify specific needs through a quantitative methodology using questionnaires. Starting from the OH Framework approach, in its heuristic form, we established the conceptual framework that is the central foundation of this study (Figure 1). We sought to explore the relationships between (i) individual factors, in which we included Resilient Coping and Self-efficacy, (ii) organizational factors, with an emphasis on Psychosocial Risk Factors (PRFs) as factors in the content or context of work that can generate OS, (iii) contextual factors, namely experiences associated with the Covid-19 pandemic, and (iv) Health and Well-being factors, highlighting Quality of Life (QoL), Distress and Health Factors in this domain (Figure 1).

Data was collected using the following assessment tools: Brief Resilient Coping Scale (EBCR), Copenhagen Psychosocial Questionnaire (COPSOQ III), Kessler Psychological Distress Scale (K10), and Sociodemographic Questionnaire. This selection focused on alignment with the OH Framework, and seeking a balance between the reliability of the measures and the number of items included, to ensure the feasibility of application, without compromising the quality of the data collected.

#### 2.3.1 Brief resilient coping scale

The BRCS is a unidimensional instrument whose scores indicate the level/capacity of coping with stress in an adaptive way (resilience). Both the original version (Sinclair and Wallston, 2004) and the Portuguese version (Pais Ribeiro and Morais, 2010), have four self-report items that are scored on five Likert levels ranging from 1 to 5 (i.e., from “almost never” to “almost always”), indicating the frequency of use of coping strategies. Results are interpreted by summing the scores of the items, which range in magnitude from 4 to 20. Reference cut-off points are used to interpret the results, with scores below 13 indicating low resilience and scores above 17 indicating high resilience (Pais Ribeiro and Morais, 2010). The original scale had an internal consistency of *α* = ~0.70 (Sinclair and Wallston, 2004), and the Portuguese version presents internal consistency of α = 0.53 (Pais Ribeiro and Morais, 2010). In the present work, α = 0.849 was verified.

#### 2.3.2 Copenhagen psychosocial questionnaire

The COPSOQ brings together international consensus on the suitability for assessing the most important PRFs in the workplace, systematically integrating the health impact that exposure represents (Fernandes and Pereira, 2016; Silva et al., 2011). Since its original development (Kristensen et al., 2005), the tool has already been revised, with COPSOQ III being the most recent version (Burr et al., 2019), whose medium version is adapted for the Portuguese population (Cotrim et al., 2022). This version consists of 85 items divided in 31 PRFs grouped into 7 main dimensions, namely Work demands, Work organization and content, Social relations and leadership, Work-individual interface, Social capital, Personality (more specifically self-efficacy), as well as a dimension related to Health and well-being indicators. The COPSOPQ III is a self-administered questionnaire using a Likert scale from 1 to 5, from “never” to “always,” indicating the agreement/frequency of issues/situations related to PRFs in the workplace. In accordance with the previous version of Silva et al. (2011), each factor can be interpreted according to the health impact that the exposure represents. To this end, starting from the cut-off points of 2.33 and 3.66, we obtain a tripartite division of the results, whose interpretation is made in terms of the percentage of responses for each level considered, grouped in a “*traffic light model*” (*green = Favorable Situation, yellow = Intermediate, and red = Health Risk Exposure*). The preliminary Portuguese version shown good reliability coefficients (Cotrim et al., 2022). In present work, internal consistency ranged between α = 0.572 and α = 0.955.

#### 2.3.3 Kessler psychological distress scale

The K10 is a brief, easy-to-use tool consisting of only 10 questions designed to assess the level and severity of a set of symptoms underlying the construct of psychological distress (Brooks et al., 2006; Kessler et al., 2002; Stolk et al., 2014). Since its original development (Kessler et al., 2002), the instrument has been adapted for numerous countries, including Portugal (Pereira et al., 2019). Responses are obtained using a Likert scale ranging from 1 to 5, indicating the frequency of psychological distress symptoms experienced in the last 30 days (i.e., from ‘no days’ to ‘every day’). The scores result from the sum of the total scale (ranging from 10 to 50), whose interpretation considers that values between 10 and 15 represent no or low distress, 16 to 21 moderate distress, 22 to 29 high distress, and 30 to 50 very high distress. Overall, scores ≥22 (high and very high distress) are considered to correspond to symptoms that are clinically significant, i.e., distress levels represent a possible impact on health/risk of developing an associated disorder. The Portuguese version showed good internal consistency with α = 0.926. In the present study, α = 0.942 was verified.

#### 2.3.4 Sociodemographic questionnaire

The Sociodemographic questionnaire applied was developed by researchers with the aim of collecting information on demographic characteristics (e.g., age, gender) and characteristics of professional activity. A question on quality of life (QoL) was also included and answered on a 1–5 Likert scale (“very bad,” “bad,” “neither good nor bad,” “good,” “very good”). Information was collected on the specificities of the impact of the Covid-19 pandemic (contextual factors), a sample checklist was also included, which seeks to gather information about feelings and behaviors associated with the COVID-19 pandemic (henceforth referred to as FAS-19, short for *COVID-19 Fears and Anxiety Scale*, as postulated by its original authors^2^). The responses are obtained using a Likert scale ranging from 1 (which corresponds to “never”) to 5 (which corresponds to “always”). In addition to analyzing the individual score for each question, the total score for the checklist was calculated, as well as the scores for each of the four groups of questions, which can be divided into (i) fear and anxiety at the individual level, (ii) daily causes of fear, (iii) positive emotions (that may be present), and (iv) lifestyle (satisfaction with it). A question about change in working modality (teleworking) was also included.

### 2.4 Procedures

A non-probabilistic sampling technique was used to recruit participants (for online, voluntary, and confidential participation). All data was collected between November 2022 and May 2023, meaning that information regarding the impact of the pandemic was collected retrospectively, i.e., in the post-pandemic phase. The request to distribute the survey form (for voluntary and confidential online participation) was made through universities, colleges, and departmental units across the country, where HE professors were sought (for voluntary and confidential online participation), using a non-probability sampling technique. The study was also publicized through HE professors’ contact networks, social media, and institutional media to recruit through snowball sampling. Reinforcements were made in terms of dissemination, considering that the minimum overall samples and quotas were met. All data was collected between November 2022 and May 2023, meaning that information regarding the impact of the pandemic was collected retrospectively, i.e., in the post-pandemic phase.

In the online form, participants could access without having to register or provide any identifying information, using LimeSurvey® software (version 3.21.1 + 191,210). This version allowed us to add a survey policy requiring informed consent prior to participation. The study was conducted according to the guidelines of the Declaration of Helsinki and approved by (*information and reference omitted for revision proposals*).

### 2.5 Data analysis

Statistical analysis was carried out using the Statistical Package for the Social Sciences software (IBM SPSS Statistics®, version 29). A descriptive analysis was carried out using measures of central tendency and dispersion (mean and standard deviation), and the qualitative / nominal variables were the subject of frequency calculations. In the process of statistical inference, the relationship between the variables was explored using Pearson’s correlation tests, with values of ±0.1 representing a low effect, ±0.3 a moderate effect and ±0.5 a high or strong effect, in addition to considering their statistical significance (probability of the correlation differing from zero, *p* ≤ 0.05; *p* ≤ 0.001) (Field, 2009).

One-way ANOVA analyses were also performed to compare more than two groups, whose effect sizes were measured using the Omega-squared Fixed-effect (*Ꞷ*^2^), considering that values of 0.01, 0.06, and 0.14 represent small, medium, and large effects, respectively (Field, 2009). In all comparisons between groups, the minimum n per group assumption was verified, to ensure a minimum corresponding to α of 0.05, test power of 0.80 (minimum recommended). A Bonferroni *post hoc* test was selected because it is more conservative and robust for groups of different sizes (Aliaga and Gunderson, 2006; Field, 2009).

Given the interest in exploring predictive models of Health and Well-being and health, several multiple regressions were used, with a method based on mathematical criteria, namely stepwise, whose option to insert and remove predictors depended on the significance (probability) of the *F* value, set at 0.05 and 0.051, respectively. The acceptability of the regression results was preceded by the preparation of the variables, specifically, the transformation of categorical variables into dummy variables, as well as the verification of prerequisites, i.e., minimum n per independent variable, independence of residuals using the Durbin-Watson test, collinearity statistics, assumptions of normality of residuals, and verification of outliers (standardized residuals with an absolute value outside the range ±3.29), as well as analysis of Cook’s distance to verify whether the values corresponded to >1.

## 3 Results

In order to facilitate the integration of the different results, it is important to note that they will be structured within the OH Framework, encompassing the respective individual, organizational, contextual, and well-being factors. In turn, the research questions were included gradually. Specifically, in response to *Question 1*, we characterized the OH Factors, constituting a descriptive exploratory approach (addressed in section 3.1). In response to *Question 2*, we analyzed the relationships between the factors analyzed (addressed in section 3.2) and analyzed the predictive effect of the various factors on well-being, from which we sought to construct the explanatory model of OS in HE teachers (addressed in section 3.2).

### 3.1 Characterization of organizational health factors

#### 3.1.1 Individual factors

In terms of resilient coping (BRCS), the responses of all respondents ranged from a minimum of 5 to a maximum of 20, with a mean slightly above the cut-off point of 13 (M = 13.96, SD = 3.52), corresponding to moderate resilient coping. The analysis of the levels of resilient coping shows some differences in the level of resilience of the total number of respondents, with 46.1% scoring low resilient coping, 28.9% scoring medium and 24.9% scoring high.

**Figure 2:**
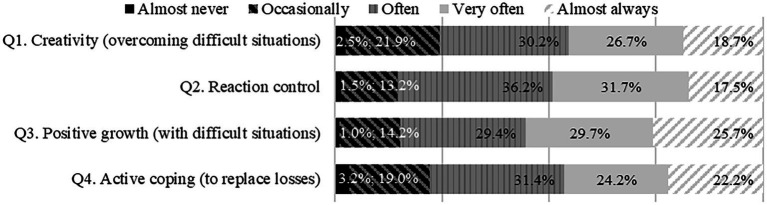
Frequency of use of coping strategies.

Considering the interpretation of the results in terms of the use of coping strategies, all the strategies included are used to a high degree, with emphasis on item Q3—Positive growth (with difficult situations) (Figure 2).

In terms of self-efficacy (using the COPSOQ III, Personality dimension), the average value was 3.86 (SD = 0.55). Subsequently, the frequency of responses in a favorable situation is quite high (65.8%). On the other hand, 32.7% of participants scored values within an intermediate situation, and only 1.5% of participants scored values classified as health impact risks.

#### 3.1.2 Organizational factors

**Figure 3:**
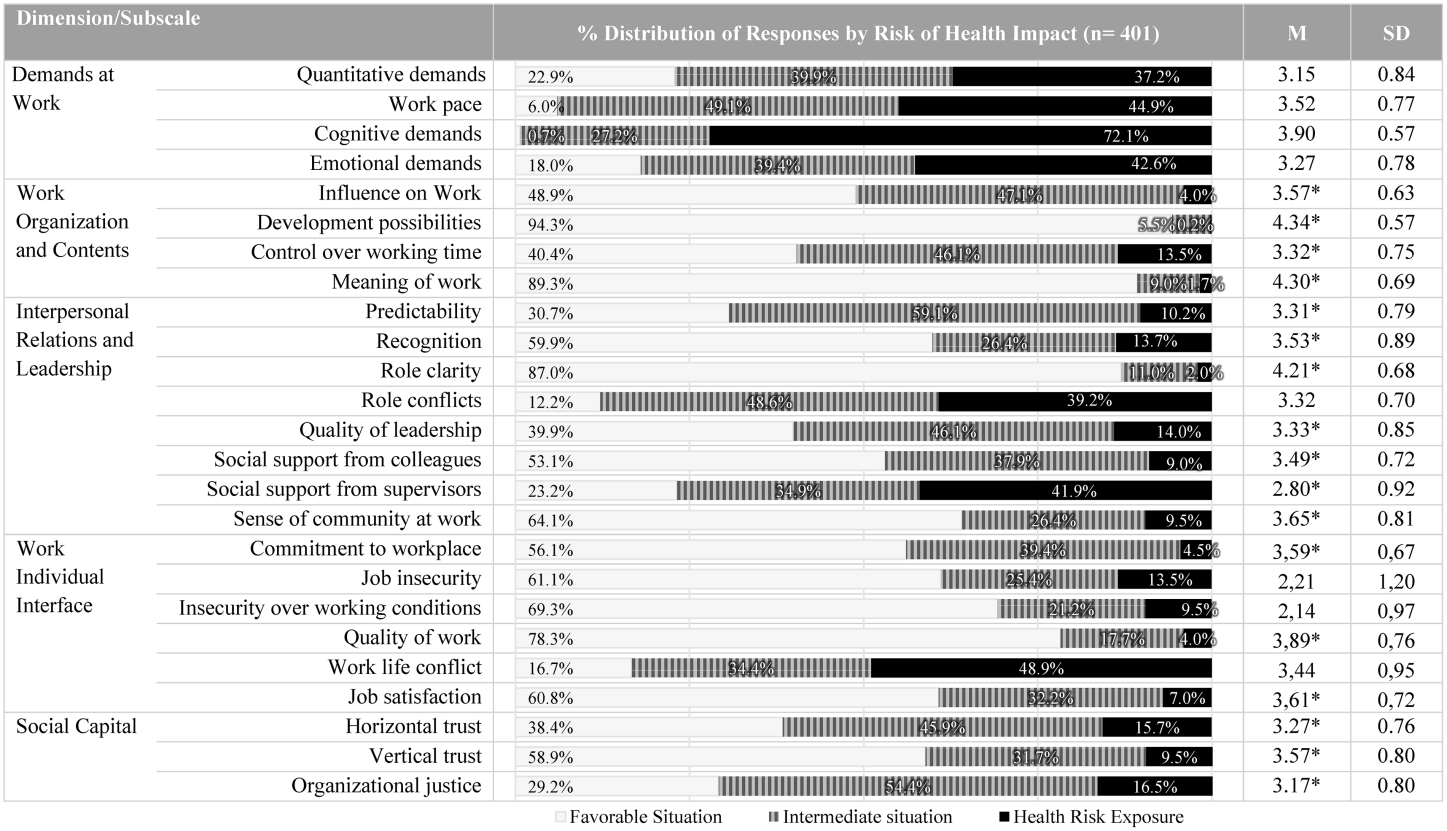
Characterization of organizational factors (PRFs). ^*^Protective factors: high values are interpreted inversely to risk, i.e., represent low risk.

Regarding the organizational factors whose values represent a risk of impact on health, the dimension of Work demands stands out (Figure 3). Particular attention should be drawn to the subscale of Cognitive Demands, where 72.1% of participants present risk of impact on health. In addition, in the subscales of Quantitative demands, Rhythm, and Emotional demands, the percentage of participants reporting exposure values with a risk of impact on health is 37.2, 44.9 and 42.6%, respectively (Figure 3). Also noteworthy is the Work–Family Conflict (underlying the Work-Individual Interface dimension), in which the percentage of participants reporting risk values is 48.9%. In addition to this, there were the PRFs of Work role conflict and social support from superiors (underlying the Social Relations and Leadership dimension), in which there was a predominance of risk situations and/or intermediate situations with an impact on health (Figure 3). In the subscale of influence at work, control over work time, predictability, quality of leadership, social support from supervisors, horizontal trust, and justice at work, there was a predominance of responses at intermediate risk, which, when added to the percentage of responses at risk, exceeded 50% (Figure 3). On the other hand, in the subscales of recognition, social support from coworkers, sense of belonging to the community, and vertical trust, the percentage of participants reporting favorable scores exceeded 50% (Figure 3). Finally, in the Development Opportunities, Meaning of Work, Transparency of Work Role, and Quality of Work, the percentage of participants reporting a favorable situation exceeded 75% (Figure 3).

#### 3.1.3 Contextual factors

**Figure 4:**
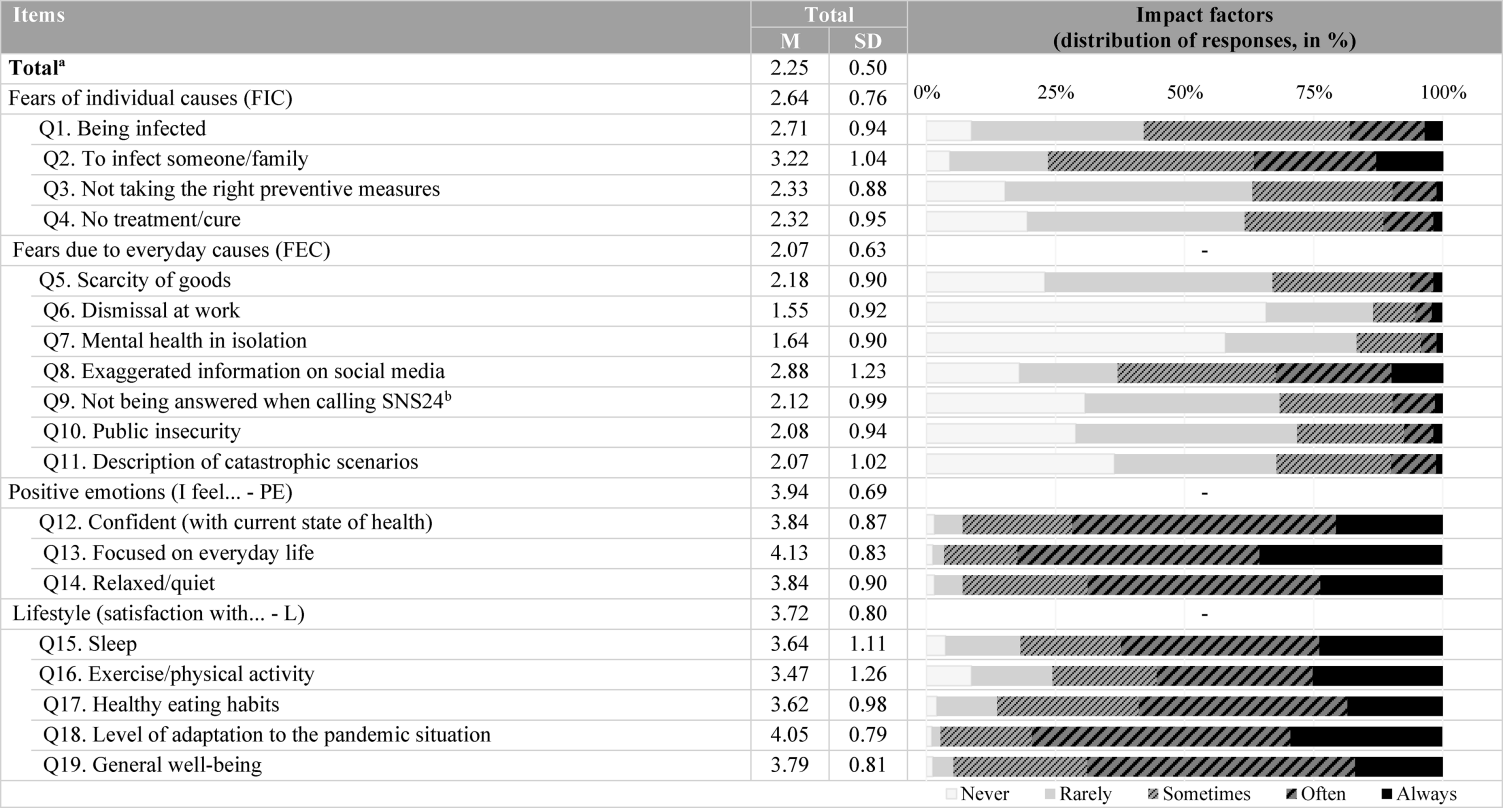
Characterization of feelings and behaviors associated with Covid-19 pandemic (FAS-19). ^a^To calculate the total score, the items in the positive dimension were reversed; ^b^SNS24 is the digital and telephone service of Portugal’s National Health Service, that offers support, triage, and referral for health-related issues, available 24 h a day. During the COVID-19 pandemic, it became the mandatory contact point for accessing healthcare services.

Regarding contextual factors, namely feelings and behaviors associated with Covid-19 pandemic (FAS-19), the average results of the total checklist are low (M = 2.25, SD = 0.50). It should be noted that the averages were higher in positive emotions (M = 3.94, SD = 0.69) and lifestyle (M = 3.72, SD = 0.80) compared to fears due to individual causes (M = 2.64, SD = 0.76), and everyday causes of fear (M = 2.07, SD = 0.63). It appears that fear of “being infected” (Q1) and of “infecting someone/family” (Q2) are the most frequent individual fears, while “exaggeration of information on social media” (Q8) is the most frequent everyday cause of fear. On the other hand, in terms of positive emotions, there is a high frequency in all areas included (Figure 4). In terms of lifestyle, however, both sleep and exercise/physical activity (Q16) show low levels of satisfaction, with responses amounting to 25% of the total sample (Figure 4).

Considering the impact of Covid-19 pandemic on changes in work modality, 82.0% (*n* = 329) of the participants switched to telework. Of these, 61.1% switched to full-time telework, and 20.7% to mixed modality (i.e., a hybrid model of on-site work and telework combined), while 0.5% did not provide information about their telework arrangements. Only a minority (*n* = 72, 17.7%) did not change their working arrangements and thus maintained on-site work.

#### 3.1.4 Health and well-being factors

**Figure 5:**
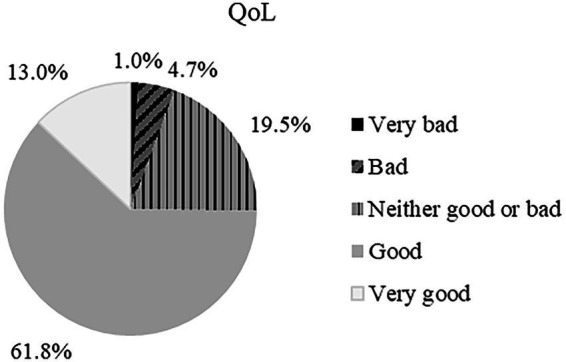
Percentage of answers considering the levels of QoL.

Regarding QoL, the response scores ranged from a minimum of 1 (“very poor”) to 5 (“very good”), with an average of 3.81 (SD = 0.75), i.e., between “neither good nor bad” and “good.” This is also supported by the distribution of responses by score, with the highest percentage of responses (61.8%) falling into the “good” QoL category (Figure 5).

Regarding distress, the responses ranged from a minimum of 10 to a maximum of 44 points, with a mean (M = 19.46, SD = 7.65) below the cut-off of 22 points, i.e., without clinical significance. Also, when interpreting the results in terms of levels of distress, the highest percentage of responses (out of the total number of respondents) fell into absent or low distress (39.2%), as well as moderate distress (28.2%). It should be noted, however, that 20.20% of the participants reported a high level of distress and 12.5% reported a very high level of distress. As such, these participants (32.7%) exhibited symptoms of psychological distress with clinical significance, i.e., the level of distress represents a possible impact on health/risk of developing an associated disorder.

**Figure 6:**
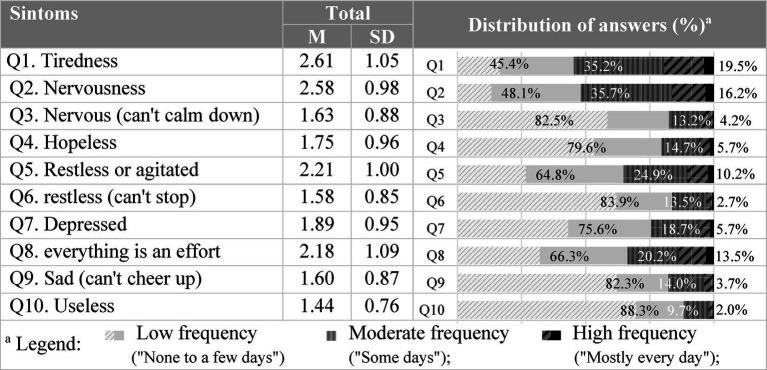
Average score and frequency of distress symptoms (experienced in the last 30 days).

Considering the interpretation of distress results in terms of symptom frequency, the highest percentage of responses falls into the low frequency category, i.e., symptoms experienced “None to a few days” (Figure 6). On the other hand, the symptoms Fatigue (Q1) and Nervousness (Q2) stand out as the most predominant in contributing to unfavorable levels of Distress. It should be noted that, in these items, the percentages corresponding to “a few days” amount to 35.2% for Q1 and 35.7% for Q2. Cumulatively, at the high frequency level (experiencing symptoms “most days to every day”), the percentages rise to 19.5 and 16.2% for the same items (Q1 and Q2, respectively) (Figure 6).

**Figure 7:**
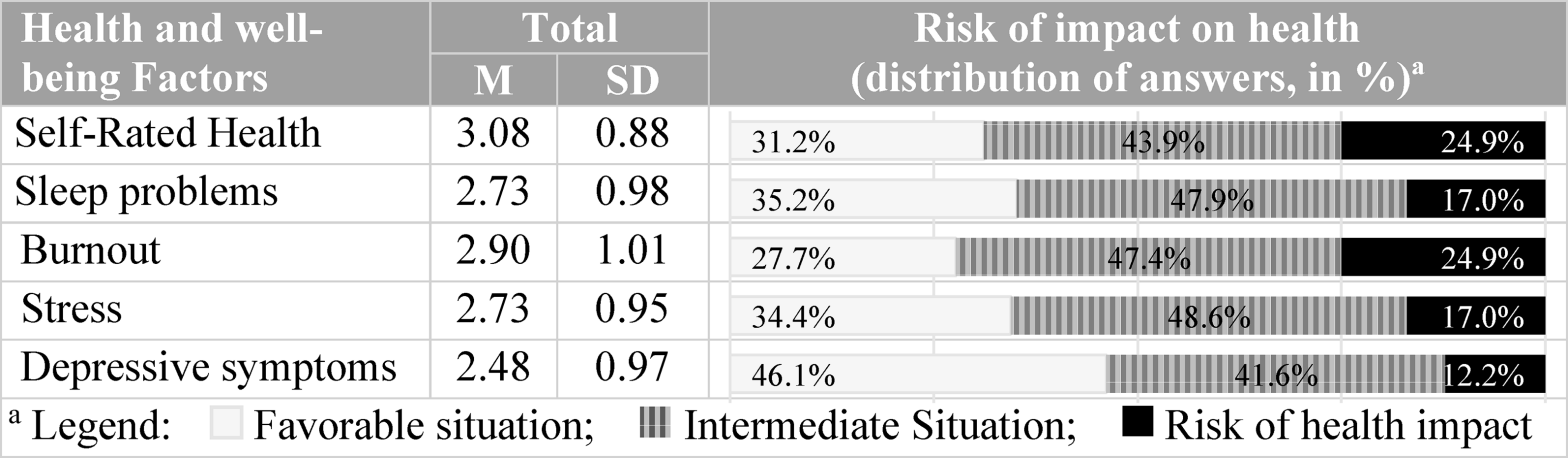
Characterization of health and well-being factors (assessed with COPSOQ III).

In terms of health factors, particularly Self-Rated Health evaluation, the mean score was 3.07 (SD = 0.88), which is classified as intermediate health risk. However, it should be noted that 24.9% of the total participants rated their Self-Rated Health as fair or poor, and these results are classified as high health risk (Figure 7).

Similarly, with respect to sleep problems, burnout, stress, and depressive symptoms, both the respective mean scores and the percentage of responses classified as having an intermediate risk of impacting health are quite high (>40%, Figure 7). It should also be noted that for the Burnout factor, the percentage of responses classified as high risk is 24.9% of all participants (Figure 7).

### 3.2 Relationship between organizational health factors

#### 3.2.1 Relationship between individual, organizational, and contextual factors

In the analysis of the relationship between individual and organizational factors, it was possible to verify multiple statistically significant correlations (Appendix 1). Particular attention is drawn to the positive significant correlations (with moderate effect) found between Resilient Coping and the PRFs of Development Opportunities (*r =* 0.356, *p* ≤ 0.01), Meaning of work (*r* = 0.416, *p* ≤ 0.01), Quality of work (*r* = 0.313, *p* ≤ 0.01), and Job satisfaction (*r* = 0.335, *p* ≤ 0.01) (Appendix 1). In terms of self-efficacy, there are positive significant correlations with the PRFs of Meaning of work (*r* = 0.337, *p* ≤ 0.01), Predictability (*r* = 0.301, *p* ≤ 0.01), Recognition (*r* = 0.306, *p* ≤ 0.01), Transparency of the work role (*r* = 0.388, *p* ≤ 0.01), Sense of belonging to the work community (*r* = 0.348, *p* ≤ 0.01), Quality of work (*r* = 0.342, *p* ≤ 0.01), and Job satisfaction, considering that most of these factors belong to the dimension of Work Relationships and Leadership (*r* = 0.300 *p* ≤ 0.01).

With regard to the relationship between individual and contextual factors, specifically the feelings and behaviors associated with Covid-19 pandemic (FAS-19 checklist), particular attention is drawn to the negative significant correlations (with moderate effect), found between Resilient Coping and total values of FAS-19 (*r* = −0.360, *p* ≤ 0.01), as well as the significant positive correlations between Resilient Coping and the dimensions of Positive Emotions (*r* = 0.362, *p* ≤ 0.01), and Lifestyle (*r* = 0.429 *p* ≤ 0.01).

Regarding the relationship between organizational and contextual factors (Appendix 1), there is a significant positive correlation (with moderate effect), with the PRFs of Job Insecurity (*r* = 0.405, *p* ≤ 0.01), Insecurity about working conditions (*r* = 0.401, *p* ≤ 0.01), and Work–family conflict (*r* = 0.379, *p* ≤ 0.01) with FAS-19 total scores. In turn, fears due to everyday factors also have a positive, significant correlation with the PRFs of Job insecurity (*r* = 3.99, *p* ≤ 0.01), and Insecurity about working conditions (*r* = 0.411, *p* ≤ 0.01). The highest number of correlations, in general, are found in the Lifestyle category. However, these are mostly of low effect, with a moderate effect only observed in the correlation with the Work–Family Conflict (*r* = −0.374, *p* ≤ 0.01).

In turn, when comparing the groups corresponding to the different work arrangements, no statistically significant differences were found between the means of the individual factors assessed, namely Resilient Coping and Self-Efficacy. However, when comparing the work modality groups in terms of the PRFs evaluated (Table 1), statistically significant differences were found only in the PRFs of Quantitative Demands [*F*_(2, 396)_ = 3.189, *p* ≤ 0.05, *Ꞷ*^2^ = 0.011], Job insecurity [*F*_(2, 396)_ = 4.727, *p* ≤ 0.01, *Ꞷ^2^* = 0.018], and Work–family conflict [*F*_(2, 396)_ = 5.243, *p* ≤ 0.01, *Ꞷ^2^* = 0.021], with low effect size.

**Table 1:** Comparison of mean PRFs between groups of work modalities.

Scale/ Dimension/ Sub-scale	Work Modality	M	SD	Comparison between groups^(a)^
Quantitative requirements	On-site (full-time, *n* = 71)	2.94	0.89	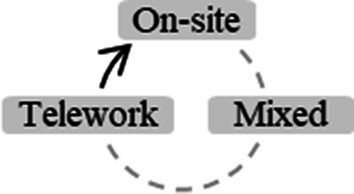
	Mixed modality^(b)^ (*n* = 83)	3.12	0.88	
	Telework (full-time, *n* = 245)	**3.22**	0.80	
Job insecurity	On-site	1.87	1.11	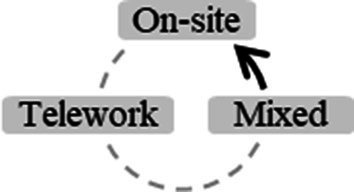
	Mixed modality	**2.46**	1.35	
	Telework	2.21	1.15	
Work–family conflict	On-site	3.13	0.99	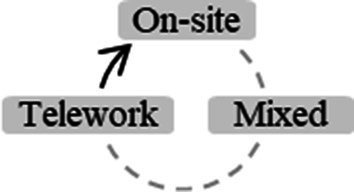
	Mixed modality	3.44	0.92	
	Telework	**3.54**	0.93	

(a) Legend: ➔ Statistically significant difference (highest to lowest mean). -- No statistically significant difference.

(b) Mixed modality = a hybrid model of on-site work and telework combined.

Bold values: highest averages.

#### 3.2.2 Relationship between the different factors with health and well-being

When analyzing the relationship between individual factors with Health and well-being factors, it is possible to see statistically significant positive and negative correlations between all the variables (Table 2). Particular attention is drawn to the positive, significant correlations of moderate effect between Resilient Coping and QoL and Self-Rated Health, as well as significant negative correlations with Distress, Sleep Problems, Burnout, Stress, and Depressive Symptoms. Self-efficacy was negatively related to Distress and positively related to Self-Rated Health (Table 2).

**Table 2:** Relationship between individual, organizational and contextual factors with well-being factors.

Dimension/subscale			Health and Well-being Factors						
			**QoL**	**Distress**	**Self-Rated Health**	**Sleep problems**	**Burnout**	**Stress**	**Depressive symptoms**
Individual factors	Resilient Coping		0.323**	−0.349**	0.360**	−0.278**	−0.313**	−0.366**	−0.389**
	Self-efficacy		0.273**	−0.315**	0.321**	−0.218**	−0.239**	−0.299**	−0.223**
Organizational Factors	Demands at Work	Quantitative demands	−0.242**	0.359**	−0.238**	0.283**	0.389**	0.351**	0.270**
		Work pace	−0.219**	0.307**	−0.167**	0.234**	0.359**	0.297**	0.201**
		Cognitive demands	−0.150**	0.162**	−0.127*	0.116*	0.192**	0.136**	0.108*
		Emotional demands	−0.157**	0.301**	−0.159**	0.255**	0.366**	0.329**	0.303**
	Work Organization and Contents	Influence on Work	0.204**	−0.197**	0.213**	−0.125*	−0.197**	−0.210**	−0.194**
		Development possibilities	0.100*	−0.130**	0.127*	−0.111*	−0.106*	−0.159**	−0.208**
		Control over working time	0.159**	−0.142**	0.217**	−0.223**	−0.196**	−0.152**	−0.126*
		Meaning of work	0.341**	−0.337**	0.322**	−0.242**	−0.269**	−0.293**	−0.354**
	Interpersonal Relations and Leadership	Predictability	0.254**	−0.205**	0.270**	−0.229**	−0.230**	−0.264**	−0.244**
		Recognition	0.337**	−0.269**	0.284**	−0.213**	−0.235**	−0.319**	−0.302**
		Role clarity	0.230**	−0.252**	0.232**	−0.178**	−0.246**	−0.266**	−0.252**
		Role conflicts	−0.167**	0.196**	−0.028	0.106*	0.211**	0.206**	0.199**
		Quality of leadership	0.227**	−0.166**	0.195**	−0.145**	−0.193**	−0.235**	−0.231**
		Social support from colleagues	0.344**	−0.172**	0.298**	−0.224**	−0.180**	−0.224**	−0.240**
		Social support from supervisors	0.242**	−0.115*	0.238**	−0.127*	−0.096	−0.190**	−0.132**
		Sense of community at work	0.348**	−0.294**	0.302**	−0.275**	−0.264**	−0.327**	−0.345**
	Work Individual Interface	Commitment to workplace	0.187**	−0.062	0.159**	−0.085	−0.076	−0.121*	−0.154**
		Job insecurity	−0.166**	0.322**	−0.263**	0.220**	0.263**	0.349**	0.288**
		Insecurity over working conditions	−0.197**	0.296**	−0.256**	0.304**	0.283**	0.364**	0.330**
		Quality of work	0.151**	−0.126*	0.120*	−0.045	−0.065	−0.132**	−0.144**
		Work life conflict	−0.309**	0.438**	−0.314**	0.327**	0.472**	0.437**	0.364**
		Job satisfaction	0.341**	−0.314**	0.390**	−0.282**	−0.277**	−0.286**	−0.335**
	Social Capital	Horizontal trust	0.259**	−0.250**	0.221**	−0.244**	−0.198**	−0.278**	−0.268**
		Vertical trust	0.270**	−0.180**	0.231**	−0.196**	−0.146**	−0.220**	−0.212**
		Organizational justice	0.306**	−0.209**	0.213**	−0.225**	−0.227**	−0.272**	−0.269**
Contextual factors	FAS-19	(Total)	−0.322**	0.539**	−0.384**	0.451**	0.520**	0.515**	0.437**
		FIC	−0.04	0.264**	−0.135**	0.201**	0.270**	0.280**	0.196**
		FEC	−0.144**	0.363**	−0.144**	0.264**	0.311**	0.349**	0.270**
		PE	0.253**	−0.377**	0.299**	−0.269**	−0.326**	−0.335**	−0.329**
		L	0.445**	−0.489**	0.499**	−0.493**	−0.525**	−0.458**	−0.426**

*ρ ≤ 0.05 and **ρ ≤ 0.01. FIC, Fears of individual causes; FEC, Fears due to everyday causes; PE, Positive Emotions; L, Lifestyle.

Bold values: moderate or strong correlations.

In the analysis of the relationship between organizational factors and Health and Well-being factors, it is possible to see statistically significant positive and negative correlations between all the variables (Table 2), with particular regard to the many statistically significant positive and negative correlations, with moderate effect, between the dimension of work-individual interface and Health and Well-being factors (Table 2), highlighting the correlations between PRF of Work–family Conflict with all, namely QoL (*r* = −0.309, *p* ≤ 0.01), Distress (*r* = 0.438, *p* ≤ 0.01), Self-Rated Health (*r* = −0.314, *p* ≤ 0.01), Sleep problems (*r* = 0.327, *p* ≤ 0.01), Burnout (*r* = 0.472, *p* ≤ 0.01), Stress (*r* = 0.437, *p* ≤ 0.01), and Depressive symptoms (*r* = 0.364, *p* ≤ 0.01).

In the analysis of the relationship between contextual factors and Health and Well-being factors, it was found significant positive and negative correlations between all the variables (Table 2), highlighting the strong correlations between FAS-19 total values and Distress (*r* = 0.539, *p* ≤ 0.01), Burnout (*r* = 0.520, *p* ≤ 0.01), and Stress (*r* = 0.515, *p* ≤ 0.01), as well as the respective FAS-19 Lifestyle dimension with burnout (*r* = −0.525, *p* ≤ 0.01).

With regard to the Health and Well-being factors assessed, compared between the work modality groups (Table 3), statistically significant differences were found in the mean values of Distress [*F*_(2, 396)_ = 4.771, *p* ≤ 0.01, *Ꞷ^2^* = 0.019], Burnout [*F*_(2, 396)_ = 4.016, *p* ≤ 0.05, *Ꞷ^2^* = 0.015], Stress [*F*_(2, 396)_ = 5.333, *p* ≤ 0.01, *Ꞷ^2^* = 0.021], and Depressive symptoms [*F*_(2, 396)_ = 4.765, *p* ≤ 0.01, *Ꞷ^2^* = 0.019], all with low effect size.

**Table 3:** Comparison of wellbeing factor means between work modality groups.

**Dimension**		**M**	**SD**	**Comparison between groups** ^**(a)**^
Distress	On-site (full-time, *n* = 71)	17.01	7.54	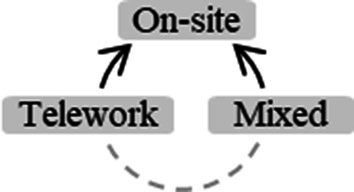
	Mixed modality^(b)^ (*n* = 83)	**20.48**	6.83	
	Telework (full-time, *n* = 245)	19.84	7.82	
Burnout	On-site	2.59	1.10	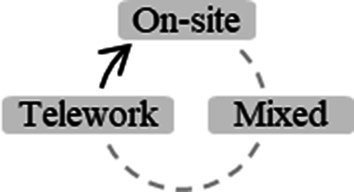
	Mixed modality	2.96	0.94	
	Telework	**2.96**	0.99	
Stress	On-site	2.41	0.96	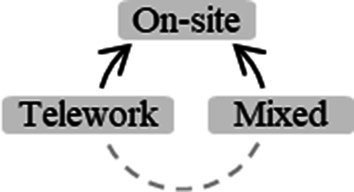
	Mixed modality	2.86	0.88	
	Telework	2.78	0.95	
Depressive symptoms	On-site	2.16	1.01	
	Mixed modality	2.56	0.96	
	Telework	2.55	0.95	

(a) Legend: ➔ Statistically significant difference (highest to lowest mean). -- No statistically significant difference.

(b) Mixed modality = a hybrid model of on-site work and telework combined.

Bold values: highest averages.

### 3.3 Predictive effect of the various factors on health and well-being

In order to verify whether factors can predict Health and Well-being factors, multiple linear regressions were applied, gradually including different predictors in three analysis groups. The comparison between the different analyses showed that the best predictive model results from the inclusion of all the factors, namely individual factors (Resilient Coping, Self-efficacy), organizational factors (PRFs), as well as contextual factors (teleworking and FAS).

The resulting predictive models and predictive variables are distinct, depending on the welfare factors included as dependent variables (Appendix 2). We also sought to study the individual effect of each variable in order to test whether the effect of a predictor on the variable differs depending on a possible third variable that serves as a proxy, which requires the joint effect to be different from the combination of the most effects considered separately (VanderWeele, 2009). The different models will be described considering the respective representative equation y = *B*_0_ + *B*_i_._(_*x*_i)_ + *B*_j_._(_*x*_j)_.^3^

For QoL, the analysis resulted in a statistically significant model [*F*(8, 392) = 22.602, *p* ≤ 0.001], which explains 31.6% of it (*R^2^* = 0.316), represented by the equation [QoL = 3.236–0.239(FAS) + 2.43(Meaning of work) − 0.207(Cognitive demands) + 0.039(Resilient Coping) + 0.187(Telework) + 0.186(Social support from colleagues) − 0.179(Development possibilities) + 0.107(Recognition)]. It should be noted that when studying the effect of each variable separately, all the variables included remained significant predictors of QoL. However, Development Possibilities separately also resulted in a statistically significant model [*F*(1, 399) = 4.006 *p* ≤ 0.05, *R^2^* = 0.010], with a positive effect (*β* = 0.100, *p* ≤ 0.05), i.e., [QoL = 3.234 + 0.133(Development possibilities)].

The statistically significant model for Distress [*F*_(6, 394)_ = 51.881, *p* ≤ 0.001] explains 44.1% of this variable (*R^2^* = 0.441), represented by the equation [Distress = 8.276 + 4.860(FAS) − 1.958(Meaning of work) + 1.466(Work–family conflict) + 1.540(Rhythm) + 0.817(Job insecurity) − 0.256(Job insecurity Resilient *Coping*)]. It should be noted that when studying the effect of each variable separately, all the variables included remained significant predictors of Distress.

With regard to Self-Rated Health, the statistically significant model [*F*_(8, 392)_ = 25.077, *p* ≤ 0.001], explains 33.9% of this variable (*R^2^* = 0.339), and is represented by [Self-Rated Health = 2.517 + 0.291(Job satisfaction) − 0.270(FAS) + 0.046(Resilient *Coping*) − 0.225(Cognitive demands) + 0.140(Social support from colleagues) − 0.077(Job insecurity) − 0.134(Work–family conflict) + 0.146(Role Conflict)]. It should be noted that in the study of the effect of each variable separately, the Role Conflict PRF, when considered in isolation, does not constitute a predictor with a significant effect on Self-Rated Health [*F*_(1, 399)_ = 0.317 *p* > 0.05, *R^2^* = 0.001]. The other variables maintained their significant effect as predictors of Self-Rated Health.

In turn, the statistically significant model for Sleep Problems [*F*_(8, 392)_ = 22.771, *p* ≤ 0.001], explains 31.7% of this variable (*R^2^* = 0.317), explained by [Sleep Problems = 1.338 + 0.567(FAS) − 0.201(Job satisfaction) + 0.140(Rhythm) + 0.102(Insecurity about working conditions) − 0.157(Horizontal trust) + 0.195(Quality of work) − 0.036(Resilient *Coping*) + 0.120(Quantitative requirements)]. It should be noted that Quality of work PRF, when considered in isolation, is not a predictor with a significant effect on Sleep problems [*F*_(1, 399)_ = 0.813 *p* > 0.05, *R^2^* = 0.002]. The other variables remained significant as predictors.

As for Burnout, the model was statistically significant [*F*_(8, 392)_ = 40.379, *p* ≤ 0.001] and explains 45.2% of this variable (*R^2^* = 0.452), encompassing [*Burnout* = 0.587 + 0.624(FAS) + 0.102(Work–family conflict) − 0.194(Meaning of work) + 0.200 (Rhythm) + 0.178 (Emotional demands) + 0.212(Quality of work) − 0.038(Resilient *Coping*) − 0.144(Job satisfaction)]. It should be noted that Quality of work, when considered in isolation, is not a significant predictor of Burnout [*F*_(1, 399)_ = 1.704 *p* > 0.05, *R^2^* = 0.004]. The other variables remained significant as predictors.

The statistically significant Stress [*F*_(7, 393)_ = 44.444, *p* ≤ 0.001] explains 44.2% of this variable (R^2^ = 0.442), whose equation is represented as [Stress = 1.230 + 0.416(FAS) + 0.188(Work–family conflict) − 0.052(Resilient *Coping*) + 0.151(Job insecurity) − 0.151 (Sense of belonging to the work community) + 0.137(Emotional demands) + 0.120(Rhythm)]. It should be noted that when studying the effect of each variable separately, all the variables included remained significant predictors of stress.

In terms of depressive symptoms, the model was statistically significant [*F*_(8, 392)_ = 30.542, *p* ≤ 0.001] and explains 38.4% of this variable (*R^2^* = 0.384), which consists of [Depressive symptoms = 1.971–0.337(FAS) − 0.205 (Meaning of work) + 0.196 (Emotional demands) − 0.060 (Resilient *Coping*) + 0.163(Work–family conflict) + 0.110(Job insecurity) − 0.172(Sense of belonging to the work community) + 0.169(Self-efficacy)]. It should be noted that Self-efficacy, when considered separately, also resulted in a statistically significant model [*F*_(1, 399)_ = 20.783 *p* ≤ 0.01, *R^2^* = 0.050], whose effect is negative (*β* = −0.223, *p* ≤ 0.01), i.e., [Depressive symptoms = 3.995–0.393(Self-efficacy)]. All the other variables included remained significant predictors.

In addition, we sought to investigate in more detail which dimensions of Fears and Anxiety Related to the Covid-19 Pandemic are capable of predicting Health and Well-being factors. To this purpose, multiple linear regression was applied, including the respective dimensions of Fears of Individual Causes, Fears of Everyday Causes, Positive Emotions, and Lifestyle. The analysis resulted in statistically significant predictive models for QoL [*F*_(1, 399)_ = 98.704, *p* ≤ 0.001, *R^2^* = 0.198], *Distress* [*F*_(3, 397)_ = 60.568, *p* ≤ 0.001, *R^2^* = 0.314], Self-Rated Health [*F*_(1, 399)_ = 132.344, *p* ≤ 0.001, *R^2^* = 0.249], Sleep Problems [*F*_(2, 398)_ = 72.647, *p* ≤ 0.001, *R^2^* = 0.267], *Burnout* [*F*_(3, 397)_ = 62.742, *p* ≤ 0.001, *R^2^* = 0.322], Stress [*F*_(3, 397)_ = 51.800, *p* ≤ 0.001, *R^2^* = 0.281], and depressive symptoms [*F*_(2, 398)_ = 53.770, *p* ≤ 0.001, *R^2^* = 0.213].

This analysis shows that fears about everyday causes had a predictive effect on all Health and Well-being factors except Self-Rated Health. However, it is clear that the pandemic may not have had only adverse effects on teachers’ Health and Well-being, since lifestyle stood out as a positive predictor in all areas of HE teachers’ Health and Well-being (Table 4).

**Table 4:** Predictive models of well-being, considering FAS-19.

Dimensions (Fear due…)	QoL	Distress	Self-Rated Health	Sleep problems	Burnout	Stress	Depressive symptoms
Individual causes	-	-			0.100*	0.102*	
Everyday causes	-	0.252**		0.159**	0.160**	0.215**	0.181**
Positive Emotion	-	−0.109**					
Lifestyle	0.445**	−0.370*	0.499**	−0.457**	−0.467**	−0.387**	−0.384**

*ρ ≤ 0.05 and **ρ ≤ 0.01.

Finally, in order to construct the Organizational Health Model, it was important to analyze the reciprocal effect between the variables included, that is, factors that act as predictors of Health and Well-being, but are equally impacted by reciprocal effects of Health and Well-being. To this end, multiple linear regressions were applied to verify whether Health and Well-being factors are predictive of individual and organizational factors. All resulting models are statistically significant (Appendix 3).

In summary, it was found that QoL is the dimension that has a reciprocal predictive effect in relation to more individual and organizational factors, more specifically in relation to Resilient Coping (*β* = 0.133, *p* ≤ 0.01), Meaning of Work (*β* = 0.187, *p* ≤ 0.01), as well as the PRFs included in the dimensions of Social Relations and Leadership and Social Capital. Self-Rated Health is the only Health and Well-being indicator that acts as a predictor for both individual factors, namely resilient coping (*β* = 0.191, *p* ≤ 0.01), and Self-efficacy (*β* = 0.220, *p* ≤ 0.01), as well as is the most powerful predictor for job satisfaction (*β* = 0.245, *p* ≤ 0.01). In turn, burnout has a reciprocal predictive effect in all PRFs included in the dimension of Demands at Work, as well as it is the most powerful predictor for Rhythm (*β* = 0.359, *p* ≤ 0.01), Emotional demands (*β* = 0.366, *p* ≤ 0.01), and work–family conflict (*β* = 0.312, *p* ≤ 0.01), and Role conflicts (*β* = 0.211, *p* ≤ 0.01) being the only predictor for the former. Distress acts as a reciprocal predictor for cognitive demands (*β* = 0.157, *p* ≤ 0.05), work–family conflict (*β* = 0.154, *p* ≤ 0.01), and acts as a negative predictor for self-efficacy (*β* = −0.208, *p* ≤ 0.01). Conversely, stress acts also as a negative predictor for Influence on Work (*β* = −0.146, *p* ≤ 0.01), Recognition (*β* = −0.208, *p* ≤ 0.01), Role clarity (*β* = −0.208, *p* ≤ 0.01), Quality of leadership (*β* = −0.173, *p* ≤ 0.01) and all PRFs included in the dimension of Social Capital. Stress is also the stronger predictor for Job insecurity (*β* = 0.289, *p* ≤ 0.01), and Insecurity over working conditions (*β* = 0.282, *p* ≤ 0.01). Depressive symptoms have the stronger reciprocal predictive effect on resilient coping (*β* = −0.255, *p* ≤ 0.01), Meaning of work (*β* = −0.218, *p* ≤ 0.01), Predictability (*β* = −0.152, *p* ≤ 0.01), and Sense of community at work (*β* = −0.212, *p* ≤ 0.01). Finally, the sleep problems dimension has a predictive effect on the smallest number of factors, namely Control over working time (*β* = −0.159, *p* ≤ 0.01) and Insecurity over working conditions (*β* = −0.148, *p* ≤ 0.01).

## 4 Discussion

The general objective of this study was to study OS in Portuguese HE teachers, implementing an innovative approach entailing the integrated application of OH Framework, which articulates individual, organizational and contextual factors (related to COVID-19 pandemic), and integrating these into the complex interaction with individual and organizational well-being. The research questions that guided this work were meticulously addressed, providing valuable information on the importance, benefits, and challenges associated with OS prevention. These same research questions will guide the structure of this section, in which we will integrate the discussion of the main results.

*Question 1:* How is the work context of HE teachers characterized, considering the OH Framework?

To answer this question, we studied the factors of organizational health from a descriptive approach. The main results show that the professional context of teachers has its own characteristics. More specifically, in terms of individual factors, we found low levels of resilient coping, and for organizational factors we found an high exposure to job demands, particularly cognitive demands. We recall that resilient coping refers to the ability to manage and cope with stressful situations in an adaptive manner and maintain a sense of well-being and balance (Denckla et al., 2020; Pais Ribeiro and Morais, 2010; Southwick et al., 2014). As such, this result is concerning, since the low resilience of HE teachers may increase vulnerability to harm associated with OS. In terms of Self-Efficacy, the results were favorable. It should be noted that positive perceptions in this area can also be leveraged as a buffer against the negative impact of OS or even burnout (Hirschle and Gondim, 2020; Salanova et al., 2003; Schwarzer and Hallum, 2008; Sonnentag, 2015; Stajkovic and Luthans, 1998).

As for organizational factors, all PRFs included in the Work Demands dimension, namely Quantitative demands, Rhythm, and Emotional demands stood out with risk values. This scenario is consistent with studies showing that the complexity of academic tasks, including teaching, research, and management, contributes to high levels of pressure and demands. In addition, the intensification of institutional responsibilities, associated with the need for continuous adaptation to technological and pedagogical changes, tends to significantly increase the workload perceived by faculty members (Bowering and Reed, 2021; Garcés-Delgado et al., 2023; Miller et al., 2011; Pace et al., 2021; Souto et al., 2018, 2019; Zapata, 2021). In fact, our results also show that Work–Family Conflicts and Role Conflicts are factors whose resulting risk must be considered, reinforcing the importance of organizational and individual strategies to mitigate the effects of OS (Heng et al., 2020; Lei et al., 2021; Xu et al., 2023). On the other hand, Development opportunities, Meaning of work, Transparency of the work role, and Quality of work presented favorable values, and should therefore be maintained and promoted. These are particularly relevant as potential protective factors, underscoring the importance of fostering supportive, enriching, and meaningful work environments to mitigate OS and promote well-being among HE teachers (Misra et al., 2022; Steger et al., 2012).

Of the contextual factors, considering the context of the Covid-19 pandemic, the overall results revealed multifaceted impacts. HE teachers reported changes to teleworking arrangements, and the main fears and anxieties associated with the pandemic were the fear of infecting someone and information overload on social media. These findings corroborate that the pandemic has intensified feelings of insecurity and vulnerability, especially in light of the rapid adaptation to new forms of teaching and working, as pointed out by several studies (Ahorsu et al., 2022; Brooks et al., 2020; Fitzpatrick et al., 2020; Killgore et al., 2020; Pierce et al., 2020; Winter et al., 2023). Additionally, the data reinforce that fear of contagion and exposure to alarmist content on social media were central elements of the pandemic experience among teachers, in line with research on the main factors of fear and misinformation during this period (Pennycook et al., 2020; Taylor et al., 2020). On the other hand, it is important to highlight the results found in terms of positive emotions and satisfaction with lifestyle, which show that, overall and in retrospect, adaptation to the pandemic has turned out to be positive, which constitute new data in the literature on this topic.

In terms of Health and Well-being, QoL was described as “Good”; however, for many HE teachers, Distress values reached clinical significance (32.7%), indicating a possible impact on health or risk of developing associated disorders. Special attention should be given to symptoms of fatigue and nervousness, which stood out as critical factors contributing to unfavorable Distress levels. In addition, participants rated their health as fair or poor, with high percentages of responses falling into the intermediate risk category for sleep problems, burnout, stress, and depressive symptoms (~40%). The results obtained in this study reveal that a significant proportion of HE teachers show concerning indicators of health and well-being impact. It should be remembered that the negative impact on the mental health of HE teachers is documented in the literature, highlighting the prevalence of symptoms such as exhaustion, insomnia, anxiety, and depression in demanding academic contexts (Carvajal and Guedea, 2021; Mahecha-Naranjo et al., 2023; Salim et al., 2023; Sun et al., 2011), which includes previous studies conducted in Portugal (Marrinhas et al., 2023; Souto et al., 2018, 2019; Teles et al., 2020).

In sum, we have thus listed the specific characteristics of HE teachers regarding OS and Health and Well-being, which have fundamental implications for constructing effective prevention and intervention measures, in a paradigm integrated with specific needs, as recommended (EU-OSHA, 2023b; Hassard et al., 2017; ISO, 2021b; Jain et al., 2018; Wolf and Prüss-Ustün, 2018). As such, it is recommended the adoption of a systematic approach to worker health surveillance that facilitates the early detection of signs and symptoms, which is crucial for the implementation of preventive interventions for work-related problems (Moreira et al., 2021). It should be remembered that employees are the core of an organization, and their level of well-being plays an important role in its functioning and performance (Bauer and Jenny, 2017; Cotton and Hart, 2003; Hart and Cooper, 2001; Lee et al., 2023).

*Question 2:* What is the relationship between OH Factors, and what is the effect on the Health and Well-being of HE teachers?

To answer this question, we analyzed the relationship between OH Factors, as well as the explanatory variablesof Health and Well-being (using linear regression). The main results were that the (i) Health and Well-being of HE teachers is influenced by the interaction of individual and organizational factors, and vice-versa, while both are influenced by contextual factors, namely experiences associated with the Covid-19 pandemic, and (ii) Work–family conflict and Job Demands are risk factors with a pronounced link with Health and Well-being, while Resilient Coping and Meaning of work stand out for their protective effect. Satisfaction with lifestyle concerning Covid-19 emerged as a explanatory variable in all Health and Well-being domains.

As such, these results provide insights into how individual and organizational factors can influence each other, highlighting the relationship between coping and self-efficacy with factors intrinsic to the organizational climate (PRFs), particularly in terms of demands, social relationships, and leadership, but also with the perception of job satisfaction and meaning. The latter are particularly relevant as potential protective factors, underscoring the importance of fostering supportive, enriching, and meaningful work environments to mitigate OS and promote well-being among HE teachers (Misra et al., 2022; Steger et al., 2012). We also emphasize that the relationships found between the domains of the OH Framework reinforce the importance of individual factors and factors intrinsic to the organizational climate as powerful allies, which take on significance in the individual-work interconnection. Remember that this interconnection gives rise to positive or negative experiences that constitute the first levels of analysis, which are likely to contribute to the well-being and salutogenesis of individuals and organizations (Cotton and Hart, 2003; Hart and Cooper, 2001; Kumar and Kirti, 2023; Singh and Jha, 2021). In fact, our results regarding Health and Well-being show significant correlations for all factors included (QoL, Distress, Self-Rated Health, Sleep Problems, Burnout, Stress, and Depressive Symptoms) with Resilient Coping, as well as with the PRFs of Work–Family Conflict, Emotional Demands, and Meaning of Work. As such, these results have implications for organizational dynamics and culture. Creating an environment that fosters empowerment through a culture of engagement can be an important strategy in promoting positive workplaces, contributing to a healthy, productive, and innovative workforce with greater job satisfaction and retention within the organization (Brito et al., 2023; Cameron and Robert, 2006; Chinoperekweyi et al., 2022; Wu and Zhou, 2023).

With regard to contextual factors, namely Fears and Anxiety regarding Covid-19, there is a negative relationship with Resilient Coping, which, however, is positive when considering the aspects of Positive Emotions and satisfaction with Lifestyle resulting from the pandemic. Additionally, Fears and Anxiety showed significant correlations with PRFs such as Job Insecurity and Work–Family Conflict, especially in teleworking arrangements, which also negatively influenced the perception of Safety and working conditions. Fears and anxiety related to COVID-19 as a whole also negatively influenced all domains of Health and Well-being, although there was an inverse relationship with satisfaction with lifestyle.

It should be pointed out that the work regime also had an effect on various PRFs and mental health indicators. First, there was an effect of the regime on job insecurity, particularly in telework (full or mixed), when compared to on-site group. This pattern suggests that distance from the workplace can intensify perceptions of professional vulnerability. Additionally, teleworking had higher averages in PRFs of quantitative demands and work–family conflict, indicating that geographical distance does not eliminate workloads and, on the contrary, can accentuate the overlap between personal and professional spheres. These factors could also contribute to higher levels of burnout, stress, distress, and depressive symptoms observed in teleworkers, both full-time and mixed, compared to on-site workers. Together, these findings reinforce the need to understand telework not only as a logistical alternative but as a context that reconfigures PRFs and requires specific mitigation strategies. Interventions aimed at managing workload, promoting work-family balance, and strengthening social support may be key to reducing the negative impacts identified. Also, there is the inherent challenge of integrating new technologies into the teaching-learning process, which, although it emerged more abruptly in response to COVID-19 for distance learning, is a current and ongoing challenge. The implications of technology both in the teaching-learning process and for HE teachers require careful consideration by HE institutions. There is now a need for digital literacy and training in e-pedagogy skills, which are essential for the effective integration of technologies into the teaching-learning process (Coman et al., 2020; Scherer et al., 2021; Virtanen and Parpala, 2023). At the same time, there is a growing need for support in establishing a professional identity adapted to the new context, focusing on the role of reconciliation (Scherer et al., 2021; Virtanen and Parpala, 2023), as well as the emergence of technostress and its impact on teachers’ well-being, where further development and research are also needed (Marrinhas et al., 2023; Springer et al., 2023).

**Figure 8:**
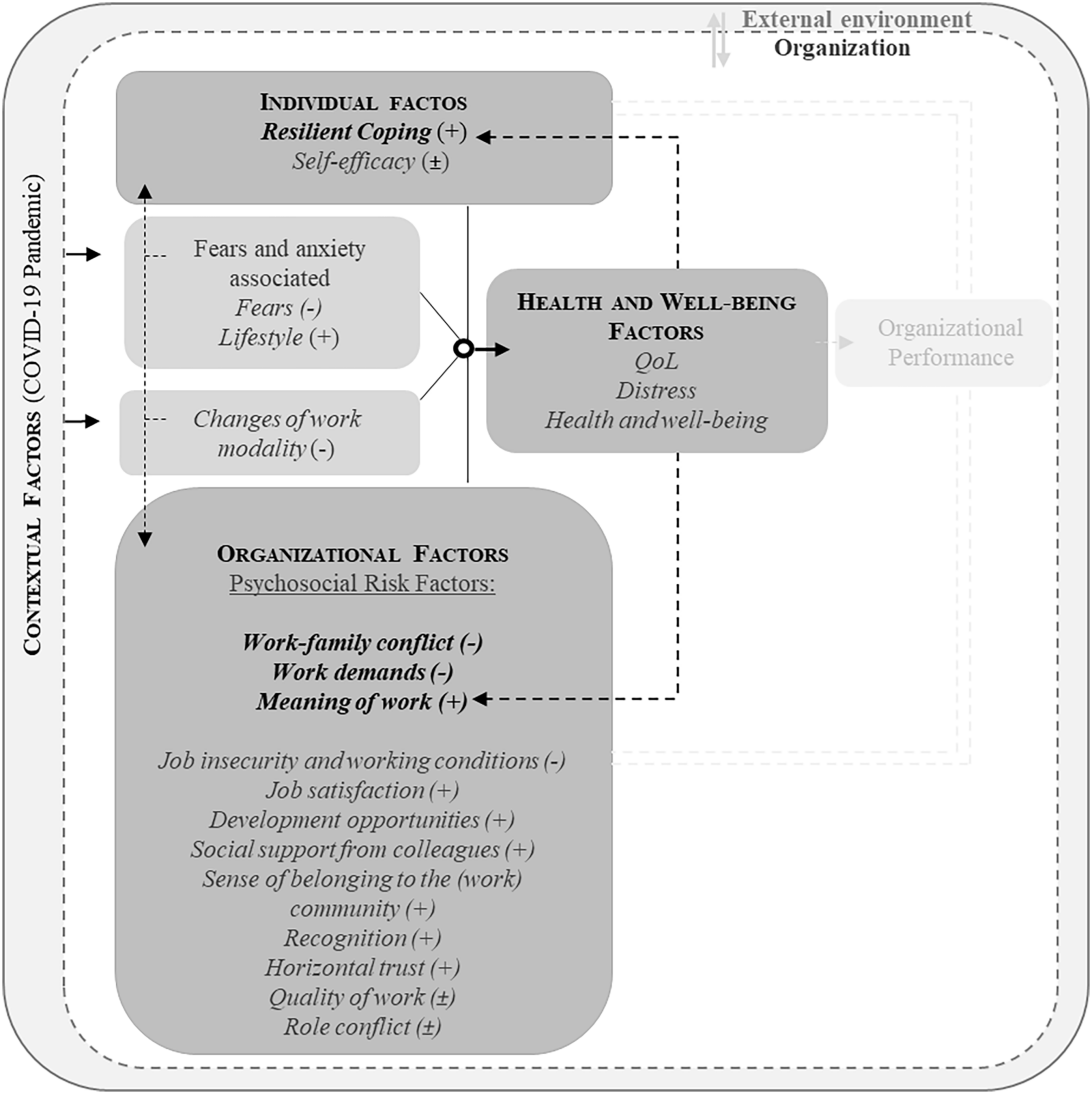
Model of organizational health in HE teachers (basic structure of the model adapted from Hart and Cooper, 2001). (−) Risk effect; (+) represents protection effect; (±) represents dual effect, i.e., when considered in isolation it has a protective effect; however, in conjunction with other predictors in the model, it may have a risk effect.

Finally, the joint analysis of individual, organizational, and contextual factors resulted in significant predictive models for the domains of Health and Well-being (Figure 8). Resilient Coping emerged as a dominant protective factor in all domains of Health and Well-being, standing out as the most relevant individual factor. Self-efficacy, on the other hand, shows a dual effect with depressive symptoms, i.e., when considered in isolation, it has a protective effect, but when combined with other co-variables in the model, it may have a risk effect (Figure 8). At the organizational level, work–family conflict and work demands were found to be risk factors for Health and Well-being, while meaningful work was found to be a protective factor. Other organizational factors such as Job insecurity, Job satisfaction, and Social support from colleagues, among others, were found to be relevant, although not common to all domains of Health and Well-being. Some of these factors, such as Job Quality and Role Conflict, had dual effects, acting as both protectors and risks, depending on the context (Figure 8). In turn, the Covid-19 pandemic proved to be impactful, but not exclusively negative, with lifestyle positively linked with Health and Well-being of HE teachers (Figure 8). And lastly, considering the reciprocal effect of OH Factors, it’s worth noting that Resilient Coping, Meaning of Work, and Health and Well-being itself, when collectively analyzed, form their own synergy of mutual influence (Figure 8).

These results, although entirely new in the literature, corroborate that worker well-being and organizational health encompass both pathogenesis and salutogenesis (Bauer and Jenny, 2017; Hart and Cooper, 2001), in the sense that they include two lines of action compatible with the general model of health development, but in this case applied to health in organizations (Bauer and Jenny, 2017; Hart and Cooper, 2001). As a result, factors relevant to pathogenesis are identified, namely risk factors, which should be targeted for intervention, as well as factors relevant to salutogenesis, i.e., protective factors, which should be promoted. The implementation of effective interventions to manage SO is essential to ensure health, productivity, and job satisfaction, and this includes assessment, prevention, intervention, and post-intervention support. On the other hand, organizations play a key role in creating an organizational climate conducive to promoting decent work and mental health at work (EC, 2011; ILO, 1999, 2019; ISO, 2021a; Koskela, 2014; Latapí Agudelo et al., 2019; ONU, 2023).

It is worth noting that the shared research findings and best practices serve as a basis for developing guidelines and standards for prevention and intervention in the workplace. However, both from a theoretical point of view and in practical intervention, OS is often seen as a subjective phenomenon, so some intervention models focus excessively on individual factors and do not consider broader organizational and systemic issues that contribute to OS, recognizing the importance of proactive and holistic approaches to mitigate exposure (Burgess et al., 2020). As such, promoting Health and Well-being requires an integrated approach that values not only individual capacity, as well as adjustment of organizational aspects, as workload management to reduce quantitative demands, work-life balance policies, such as flexible working hours and the right to disconnect, as well as a culture of engagement and continuous improvement (Bowering and Reed, 2021; Brito et al., 2023), that may be highly recommended measures to prevent and mitigate OS. On the other hand, this reinforces the idea that a holistic perspective on the relationship between the individual, the organization, and the environment is crucial for managing individual and organizational well-being (Hart and Cooper, 2001; Thanem and Elraz, 2022). Thus, the multidimensional approach adopted, with the practical application of the OH Framework, represents a significant innovation in research on OS, allowing a holistic understanding of the complex interactions between individual, organizational, and contextual factors that influence teachers’ well-being. This approach not only offers a new methodological perspective for studying OS in HE contexts, but also constitutes an unprecedented contribution to international scientific literature. By integrating dimensions that are traditionally analyzed alone, this approach allows for a more accurate identification of risk and protective factors, providing robust support for the development of intervention and health promotion strategies in teaching work. For the organization of HE, this may include access to continuing education, support in areas of expertise, and creating a clear path for career progression. At the same time, promoting a culture of engagement in collaboration, through dedicated platforms, mentoring programs, or open communication channels between ES teachers, can increase their collective effectiveness (Edinger and Edinger, 2018; Hargreaves, 2019; Tantawy, 2020), which are essential for promoting the resilience, adaptability, and well-being of ES teachers (Hargreaves, 2019). Remember that employees are the core of an organization, and their level of well-being plays an important role in its functioning and performance (Bauer and Jenny, 2017; Cotton and Hart, 2003; Hart and Cooper, 2001; Lee et al., 2023.

In a broad sense, these results reinforce the importance of HE institutions prioritizing the assessment, monitoring, and management of these factors, with a view to prevention and intervention focused on specific needs, as well as enhancing protective factors, as recommended (EU-OSHA, 2023; Hassard et al., 2017; ISO, 2021b; Jain et al., 2018; Wolf and Prüss-Ustün, 2018).

### 4.1 Limitations

This study has some limitations that should be considered when understanding the results. The exclusive use of self-administered questionnaires can introduce bias in the results. Also, given that this study was mainly an online survey, it may have attracted more participants who feel more exposed to or attracted by the topic, so the self-selection of participants is the first limitation to be listed.

Although it is very important to reflect on the particular reality and possible related factors from a cross-sectional perspective, it is recognized that the design of the study limits the ability to establish causal relationships between variables, since we can only suggest possible predictions, not prove them. Also, looking only at a work system at a given moment is limiting and does not consider the temporal context of continuous changes and adaptations. Furthermore, although the variables analyzed in this study are recognized in the literature on OS, this literature does not include a systematic integration of the conceptual model adopted. The absence of studies using the same theoretical framework hinders direct comparisons between results from different methodological approaches and may limit discussion, increasing the risk of overinterpretation or confirmation bias. This limitation also impacts the generalization of the findings.

Although the overall sample is considered representative, the non-probabilistic sampling limits the generalization of the results, so caution is needed when extrapolating conclusions to other contexts. Also, when considering the distribution of the sample across subpopulations (gender, type of education, and nature of the educational institutions), the sampling error and proportion are variable, noting that the sample is representative of the public education subpopulation (90% ≈ 4.4%), and marginally significant for the subpopulations of polytechnic education (85% ≈ 4.7%) and female gender (85% ≈ 4.5). As such, the representativeness of the different subgroups may limit the generalizability of the results, as well as the absence of comparative analyses between these subgroups is another limitation, preventing the identification of possible significant differences in OS and well-being among different teacher profiles.

Regarding construct validity, although a critical review of the instruments was conducted, it should be noted that the Portuguese version of the Brief Resilient Coping Scale shows low internal consistency (α = 0.53). This result may be common in short scales, given that traditional psychometric criteria were designed for more extensive instruments. Despite this, the literature supports the adequacy of the scale in different populations, and in the present sample, the indices were excellent (α = 0.849), reinforcing its relevance. Nevertheless, this characteristic should be considered as a limitation, as it may influence interpretations in different contexts.

Finally, it is worth noting that the collection of information on the impact of the pandemic was carried out retrospectively (in the post-pandemic phase), which may introduce memory bias, as participants may not accurately recall their experiences or perceptions during the period analyzed. This limitation should be considered when interpreting the results, as it may affect the validity of the responses.

### 4.2 Future research

The proposed research is not limited to this study but rather emerges as a multi- and interdisciplinary field. This field has several nuances and dimensions that still need to be further explored in order to obtain a complete and comprehensive understanding of the phenomena in question. We propose that priority should be given to long-term research, which allow us to observe the evolution of occupational and health factors over time, contributing to a better understanding of causal relationships, preferably with the inclusion of diversifying the information collected. It is also recommended that future studies include objective measures of stress and well-being, complementing self-reported data with physiological indicators (such as heart rate, cortisol levels, or heart rate variability) and behavioral indicators (e.g., sleep patterns or physical activity). This multi-method approach may increase the validity of the results and reduce the bias associated with the exclusive use of subjective instruments. It is also recommended to include qualitative content through action research, allowing for a more in-depth and contextualized analysis of participants’ experiences. This approach can contribute to an integrated understanding of the proposed model and underlying phenomena by combining empirical data with reflective and collaborative processes. In addition, action research favors the identification of concrete practices and intervention strategies, enriching the interpretation of quantitative results and broadening the applicability of the study.

The interaction observed between the different factors and variables analyzed in this study highlights the need for future research that explores the application of OH Framework, considering diverse institutional and cultural contexts, allowing not only to test its external validity, as well as a more holistic and integrated understanding of the phenomenon. Comparative research in international settings can expand the body of knowledge about specific work contexts, favoring the incorporation of broader cultural factors in the analysis of the dynamics associated with the causes of OS. This approach will contribute to the development of more robust explanatory models that are applicable in different realities. In addition, comparative studies involving sample subgroups based on sociodemographic and professional variables are particularly relevant for identifying differentiated patterns and contextual specificities, contributing to identifying significant variations and developing strategies that are better adapted to the diverse realities of HE.

Promoting the well-being of HE teachers requires a comprehensive approach that promotes prevention and timely intervention, as well as being tailored to specific needs, prioritizing assessment and risk management. As such, the link with monitoring and assessment systems to analyze the real-time impact of the OS experience is the first proposal in terms of future studies. More specifically, we propose the development of an e-health platform in the context of HE, incorporating an assessment system associated with new and emerging technologies. It is noted that the wide umbrella of Digital Transformation 4.0 could enable real opportunities in occupational health psychology, pushing the boundaries towards a *Psychology 4.0* (Souto et al., 2022). The continued development of this area of research in future studies could contribute significantly to the advancement of scientific thematic knowledge, as well as to the improvement of working conditions and the well-being of professionals (Hashmi and Yadav, 2018; Hickey et al., 2021; Sánchez-Reolid et al., 2020).

The validation of prevention tools, necessary for promoting the health and well-being of workers and organizations, adaptable to different contexts and cultures, is also a challenge. It is necessary to embrace the complexity and multifactorial nature of OS problems and move beyond intervention models that are overly focused on individual factors and do not consider broader organizational and systemic aspects, which, according to the literature on the subject, have limited effectiveness in addressing the underlying causes of OS (Burgess et al., 2020; Cooper, 1998; Cox and Griffiths, 2005; Quick and Quick, 1984). This highlights the contribution to the role of psychologists not only in terms of basic holistic assessment, but also for the implementation of comprehensive prevention and intervention strategies.

## 5 Conclusion

In summary, the Health and Well-being of HE teachers results from a complex interaction between individual, organizational, and contextual factors, such as the challenges posed by the pandemic and changes in working conditions. From a theoretical perspective, the study highlights the relevance of a holistic approach to analyzing the relationship between individuals, organizations, and the environment, emphasizing the adequacy of the OH Framework. The results demonstrate the need for specific preventive and intervention strategies for teachers, with a focus on mental health and institutional support. The integration of organizational policies and practices that promote a culture of Health and Well-being is essential to enable teachers to reach their full potential, contributing to more effective and impactful teaching. The prevention of OS stress and the promotion of Health and Well-being should be seen as strategic imperatives, with direct effects on the quality of teaching and the success of institutions, as well as on the development and innovation of society (Belwal et al., 2023; Ngirande, 2021; Singh and Ryhal, 2021; Valdiviezo and Guevara, 2022; Virtanen and Parpala, 2023; Wang et al., 2020; Xu et al., 2023). Finally, relevant implications for public policy are highlighted, with an emphasis on the responsibility of organizations to implement action plans for the prevention of mental illness and the promotion of health at work. Assessment should be understood as an integral part of a broader risk management process, in which mitigation at source and the guarantee of decent work are fundamental pillars (EC, 2011; ILO, 1999, 2019; ISO, 2021a; Koskela, 2014; Latapí Agudelo et al., 2019; ONU, 2023).

## Data Availability

The raw data supporting the conclusions of this article will be made available by the authors, without undue reservation.

## Footnotes

1 The DGEEC is a central service of the Portuguese Government, whose main mission is to produce, analyze, and disseminate official statistics in the areas of education, HE, science, and technology. It acts as technical support for the formulation of public policies and the strategic and operational planning of the Ministry of Education and Science.

2 Checklist developed by Pereira et al. during the COVID-19 pandemic, whose validation study is in the process of being published. – see Acknowledgments. In present work, internal consistency for total checklist was *α* = 0.667, as well as for the four groups of questions internal consistency ranged between *α* = 0.673 and *α* = 0.854.

3 y is the dependent variable, the *B*_0_ is the constant, the *B*_ij_ corresponds to the unstandardized Beta Coefficient of the variable, and the *x*_ij_ the respective variable. The predictors will be included in the equation considering their relative importance, based on their normalized Beta Coefficients (*β*) – Appendix 2. The term “predictors” refers to the explanatory variables used to estimate or predict the dependent variable, without seeking to establish cause-and-effect relationships.
