# Supplementary material for: Organizational health factors: multidimensional insights into occupational stress in higher education
Source: Front Psychol. 2026 Jan 21;16:1715167. doi: 10.3389/fpsyg.2025.1715167 (PMC12867872; doi:10.3389/fpsyg.2025.1715167)
Supplement: Supplementary file 1 [file Table_1.DOCX]

Appendix 1: Relationship between individual, organizational and contextual factors.

| Dimension/Subscale | | | Individual factors | | Contextual factors | | | | |
| --- | --- | --- | --- | --- | --- | --- | --- | --- | --- |
|  |  |  | **Resilient Coping** | **Self-efficacy** | **FAS-19** | | | | |
|  |  |  |  |  | **(Total)** | **FIC** | **FEC** | **PE** | **L** |
| Individual factors | Resilient Coping | | **-** | **-** | -.360** | -.133** | -.127* | .362** | .429** |
|  | Self-efficacy | |  | **-** | -.288** | -.141** | -.145** | .299** | .265** |
| Organizational Factors | Demands at Work | Quantitative demands | -.185** | -.253** | .264** | .183** | .119* | -.187** | -.262** |
|  |  | Work pace | 0.067 | -.128* | .176** | .136** | .156** | -0.055 | -.118* |
|  |  | Cognitive demands | .214** | -0.046 | .125* | .131** | .112* | -0.05 | -0.051 |
|  |  | Emotional demands | -0.047 | -.162** | .293** | .177** | .205** | -.189** | -.242** |
|  | Work Organization and Contents | Influence on Work | .258** | .190** | -.187** | -.112* | -0.025 | .195** | .233** |
|  |  | Development possibilities | **.356**** | .259** | -.168** | -0.044 | -0.061 | .244** | .173** |
|  |  | Control over working time | .224** | .180** | -.249** | -.172** | -0.078 | .255** | .246** |
|  |  | Meaning of work | **.416**** | **.337**** | -.163** | -0.009 | 0.007 | .195** | .288** |
|  | Interpersonal Relations and Leadership | Predictability | .193** | **.301**** | -.173** | -0.029 | -0.076 | .162** | .222** |
|  |  | Recognition | .264** | **.306**** | -.202** | -0.073 | -0.094 | .181** | .227** |
|  |  | Role clarity | .295** | **.388**** | -.232** | -0.06 | -.138** | .209** | .247** |
|  |  | Role conflicts | 0.045 | -.114* | 0.098 | .100* | 0.018 | -0.062 | -.105* |
|  |  | Quality of leadership | .204** | .174** | -0.091 | -0.021 | -0.043 | 0.081 | .109* |
|  |  | Social support from colleagues | .219** | .244** | -.177** | -0.066 | -0.011 | .209** | .251** |
|  |  | Social support from supervisors | .241** | .247** | -0.08 | -0.054 | 0.02 | 0.095 | .122* |
|  |  | Sense of community at work | .227** | **.348**** | -.196** | -0.068 | -0.041 | .227** | .252** |
|  | Work Individual Interface | Commitment to workplace | .213** | .165** | 0.056 | .111* | .148** | 0.05 | 0.086 |
|  |  | Job insecurity | -.179** | -.118* | **.405**** | .174** | **.399**** | -.224** | -.279** |
|  |  | Insecurity over working conditions | -.212** | -.153** | **.401**** | .216** | **.411**** | -.213** | -.231** |
|  |  | Quality of work | **.313**** | **.342**** | -.112* | 0.01 | -0.001 | .169** | .184** |
|  |  | Work life conflict | -.163** | -.230** | **.379**** | .207** | .231** | -.231** | **-.374**** |
|  |  | Job satisfaction | **.335**** | **.300**** | -.175** | -0.02 | -0.077 | .145** | .243** |
|  | Social Capital | Horizontal trust | 0.08 | .204** | -.150** | -.104* | -0.067 | 0.097 | .153** |
|  |  | Vertical trust | .186** | .263** | -.129** | -0.002 | -0.063 | .129** | .170** |
|  |  | Organizational justice | .170** | .259** | -.159** | -0.065 | -0.094 | .121* | .162** |

* *ρ≤* .05 e ** *ρ≤* .01.

FIC= Fears of individual causes, FEC= Fears due to everyday causes, PE= Positive Emotions, L= Lifestyle

Appendix 2: Predicted models for well-being factors.

| Dimension/Sub-scale | | | Well-being Factors | | | | | | |
| --- | --- | --- | --- | --- | --- | --- | --- | --- | --- |
|  |  |  | **QoL** | ***Distress*** | **Self-Rated Health** | **Sleep Problems** | ***Burnout*** | **Stress** | **Depressive symptoms** |
| Individual factors | *Resilient Coping* | | **.184**** | **-.118**** | **.183**** | -.129** | **-.134*** | **-.194**** | **-.216**** |
|  | Self-efficacy | | - | - | - | - | - | - | *.096* |
| Organizational Factors | Demands at Work | Quantitative demands | - | - | - | .103* | - | - | - |
|  |  | Work pace | - | .155** | - | .110* | .152** | .098* | - |
|  |  | Cognitive demands | -.155** | - | -.144** | - | - | - | - |
|  |  | Emotional demands | - | - | - | - | .137** | .112** | .156** |
|  | Work Organization and Contents | Influence on Work | - | - | - | - | - | - | - |
|  |  | Development possibilities | *-.135** | - | - | - | - | - | - |
|  |  | Control over working time | - | - | - | - | - | - | - |
|  |  | Meaning of work | .223** | -.177** | - | - | -.133** | - | -.146** |
|  | Interpersonal Relations and Leadership | Predictability | - | - | - | - | - | - | - |
|  |  | Recognition | .126* | - | - | - | - | - | - |
|  |  | Role clarity | - | - | - | - | - | - | - |
|  |  | Role conflicts | - | - | *.116** | - | - | - | - |
|  |  | Quality of leadership | - | - | - | - | - | - | - |
|  |  | Social support from colleagues | .178** | - | .114* | - | - | - | - |
|  |  | Social support from supervisors | - | *-* | *-* | *-* | *-* | *-* | *-* |
|  |  | Sense of community at work | - | - | - | - | - | -.129** | -.143** |
|  | Work Individual Interface | Commitment to workplace | - | - | - | - | - | - | - |
|  |  | Job insecurity | - | .128** | -.104* | - | - | .191** | .136** |
|  |  | Insecurity over working conditions | - | - | - | .101* | - | - | - |
|  |  | Quality of work | - | *-* | *-* | *.152*** | *.160*** | *-* | *-* |
|  |  | Work life conflict | - | .182** | -.144** | - | .190** | .189** | .159** |
|  |  | Job satisfaction | - | - | .237 | -.147** | -.103* | - | - |
|  | Social Capital | Horizontal trust | - | - | - | -.122** | - | - | - |
|  |  | Vertical trust | - | - | - | - | - | - | - |
|  |  | Organizational justice | - | - | - | - | - | - | - |
| Contextual factors | Fears and Anxiety about the Pandemic (FAS) | | **-.159**** | **.319**** | **-.153**** | **.291**** | **.311**** | **.221**** | **.174**** |
|  | Teleworking | | .121** | - | - | - | - | - | - |

*Note: The values shown correspond to normalized Beta Coefficients* (*β*), whose t-test significance is represented by * *ρ≤* .05 e ** *ρ≤* .01.

Appendix 3. Reciprocal predictive effect of well-being on individual and organizational factors.

| Dimensão/ Subescala | | | F | R^2^ | Well-being Factors | | | | | | |
| --- | --- | --- | --- | --- | --- | --- | --- | --- | --- | --- | --- |
|  |  |  |  |  | **QoL** | ***Distress*** | **Self-Rated Health** | **Sleep Problems** | ***Burnout*** | **Stress** | **Depressive Symptoms** |
| Individual factors | *Resilient Coping* | | 35.309** | .211 | .133** | - | .191** | - | - | - | -.255 |
|  | Self-efficacy | | 31.337** | .136 | - | -.208** | .220** | - | - | - | - |
| Organizational Factors | Demands at Work | Quantitative demands | 38.497** | .162 | - | .157* | - | - | .272** | - | - |
|  |  | Work pace | 58.869** | .129 | - | - | - | - | .359** | - | - |
|  |  | Cognitive demands | 15.339** | .037 | - | - | - | - | .192** | - | - |
|  |  | Emotional demands | 61.628** | .134 | - | - | - | - | .366** | - | - |
|  | Work Organization and Contents | Influence on Work | 13.354** | .063 | - | - | .151** | - | - | -.146** | - |
|  |  | Development possibilities | 5.547** | .043 | - | - | - | - | - | - | -.208** |
|  |  | Control over working time | 14.526** | .068 | - | - | .150** | -.159** | - | - | - |
|  |  | Meaning of work | 30.460** | .187 | .187** | - | .144** | - | - | - | -.218** |
|  | Interpersonal Relations and Leadership | Predictability | 16.685** | .112 | .127** | - | .146** | - | - | - | -.152** |
|  |  | Recognition | 36.494** | .155 | .295** | - | - | - | - | -.208** | - |
|  |  | Role clarity | 19.612** | .090 | .136** | - | - | - | - | -208** | - |
|  |  | Role conflicts | 18.661** | .045 | - | - | - | - | .211** | - | - |
|  |  | Quality of leadership | 16.608** | .077 | .160** | - | - | - | - | -.173** | - |
|  |  | Social support from colleagues | 33.245** | .143 | .262** | - | .177** | - | - | - | - |
|  |  | Social support from supervisors | 16.985** | .079 | .168** | - | .160** | - | - | - | - |
|  |  | Sense of community at work | 29.262** | .181 | .210** | - | .115** | - | - | - | -.212** |
|  | Work Individual Interface | Commitment to workplace | 14.401** | .035 | .187** | - | - | - | - | - | - |
|  |  | Job insecurity | 31.829** | .138 | - | - | -.141** | - | - | .289** | - |
|  |  | Insecurity over working conditions | 34.476** | .148 | - | - | - | .148** | - | .282** | - |
|  |  | Quality of work | 9.324** | .023 | .151** | - | - | - | - | - | - |
|  |  | Work life conflict | 43.753** | .248 | -.109** | .154** | - | - | .312 | - | - |
|  |  | Job satisfaction | 34.413** | .206 | .160 | - | .245** | - | - | - | -.168** |
|  | Social Capital | Horizontal trust | 23.193** | .104 | .179** | - | - | - | - | -.209** | - |
|  |  | Vertical trust | 19.334** | .089 | .217 | - | - | - | - | -.136 | - |
|  |  | Organizational justice | 27.425** | .121 | .236 | - | - | - | - | -.181 | - |

*Note: The values shown correspond to normalized Beta Coefficients (β), whose t-test significance is represented by* * *ρ≤* .05 e ** *ρ≤* .01.
